# Integrated Pathway-Based Approach Identifies Association between Genomic Regions at CTCF and CACNB2 and Schizophrenia

**DOI:** 10.1371/journal.pgen.1004345

**Published:** 2014-06-05

**Authors:** Dilafruz Juraeva, Britta Haenisch, Marc Zapatka, Josef Frank, GROUP Investigators, PSYCH-GEMS SCZ working group, Stephanie H. Witt, Thomas W. Mühleisen, Jens Treutlein, Jana Strohmaier, Sandra Meier, Franziska Degenhardt, Ina Giegling, Stephan Ripke, Markus Leber, Christoph Lange, Thomas G. Schulze, Rainald Mössner, Igor Nenadic, Heinrich Sauer, Dan Rujescu, Wolfgang Maier, Anders Børglum, Roel Ophoff, Sven Cichon, Markus M. Nöthen, Marcella Rietschel, Manuel Mattheisen, Benedikt Brors

**Affiliations:** 1 Division of Theoretical Bioinformatics, German Cancer Research Center (DKFZ), Heidelberg, Germany; 2 German Center for Neurodegenerative Diseases (DZNE), Bonn, Germany; 3 Institute of Human Genetics, University of Bonn, Bonn, Germany; 4 Federal Institute for Drugs and Medical Devices (BfArM), Bonn, Germany; 5 Department of Psychiatry, University of Bonn, Bonn, Germany; 6 Division of Molecular Genetics, German Cancer Research Center (DKFZ), Heidelberg, Germany; 7 Department of Genetic Epidemiology in Psychiatry, Central Institute of Mental Health, Medical Faculty Mannheim/Heidelberg University, Mannheim, Germany; 8 Department of Genomics, Life and Brain Center, University of Bonn, Bonn, Germany; 9 Institute for Neuroscience and Medicine (INM-1), Research Centre Juelich, Juelich, Germany; 10 National Centre for Integrated Register-based Research (NCRR), Department of Economics and Business, Aarhus University, Aarhus, Denmark; 11 Division of Molecular and Clinical Neurobiology, Department of Psychiatry, Ludwig-Maximilians-University, Munich, Germany; 12 Department of Psychiatry, University of Halle-Wittenberg, Halle, Germany; 13 Analytic and Translational Genetics Unit, Department of Medicine, Massachusetts General Hospital, Boston, Massachusetts, United States of America; 14 Broad Institute of MIT and Harvard, Cambridge, Massachusetts, United States of America; 15 Institute for Medical Biometry, Informatics, and Epidemiology, University of Bonn, Bonn, Germany; 16 Department of Genomic Mathematics, University of Bonn, Bonn, Germany; 17 Department of Biostatistics, Harvard School of Public Health, Boston, Massachusetts, United States of America; 18 Department of Psychiatry and Psychotherapy, University Medical Center Georg-August-Universität, Göttingen, Germany; 19 Department of Psychiatry, University of Bonn, Bonn, Germany; 20 Department of Psychiatry and Psychotherapy, Jena University Hospital, Jena, Germany; 21 Department of Biomedicine, Aarhus University, Aarhus C, Denmark and Center for Integrated Sequencing, iSEQ, Aarhus, Denmark; 22 Lundbeck Foundation Initiative for Integrative Psychiatric Research, iPSYCH, Aarhus and Copenhagen, Denmark; 23 Centre for Psychiatric Research, Aarhus University Hospital, Risskov, Denmark; 24 UCLA Center for Neurobehavioral Genetics, Los Angeles, California, United States of America; 25 Department of Psychiatry, Rudolf Magnus Institute of Neuroscience, University Medical Center Utrecht, Utrecht, The Netherlands; 26 Department of Medical Genetics, University Hospital Basel, Basel, Switzerland; Cardiff University, United Kingdom

## Abstract

In the present study, an integrated hierarchical approach was applied to: (1) identify pathways associated with susceptibility to schizophrenia; (2) detect genes that may be potentially affected in these pathways since they contain an associated polymorphism; and (3) annotate the functional consequences of such single-nucleotide polymorphisms (SNPs) in the affected genes or their regulatory regions. The Global Test was applied to detect schizophrenia-associated pathways using discovery and replication datasets comprising 5,040 and 5,082 individuals of European ancestry, respectively. Information concerning functional gene-sets was retrieved from the Kyoto Encyclopedia of Genes and Genomes, Gene Ontology, and the Molecular Signatures Database. Fourteen of the gene-sets or pathways identified in the discovery dataset were confirmed in the replication dataset. These include functional processes involved in transcriptional regulation and gene expression, synapse organization, cell adhesion, and apoptosis. For two genes, i.e. *CTCF* and *CACNB2*, evidence for association with schizophrenia was available (at the gene-level) in both the discovery study and published data from the Psychiatric Genomics Consortium schizophrenia study. Furthermore, these genes mapped to four of the 14 presently identified pathways. Several of the SNPs assigned to *CTCF* and *CACNB2* have potential functional consequences, and a gene in close proximity to *CACNB2*, i.e. *ARL5B*, was identified as a potential gene of interest. Application of the present hierarchical approach thus allowed: (1) identification of novel biological gene-sets or pathways with potential involvement in the etiology of schizophrenia, as well as replication of these findings in an independent cohort; (2) detection of genes of interest for future follow-up studies; and (3) the highlighting of novel genes in previously reported candidate regions for schizophrenia.

## Author Summary

Large-scale genetic studies of complex diseases such as schizophrenia have identified a variety of susceptibility loci. Since many of the respective variants have only a weak influence on disease risk, pathophysiological interpretation of the results is problematic. Investigation of the joint effects of multiple functionally related genes or pathways increases the power to detect disease related genes, and provides insights into the etiology of the disease in question. In the present study, an integrated hierarchical approach was applied to: (i) identify pathways associated with complex neuropsychiatric disease schizophrenia (ii) detect potentially affected genes in these pathways; and (iii) annotate the functional consequences of genetic markers in the affected genes or their regulatory regions. Two samples comprising >10,000 individuals of European ancestry as well as data from the Psychiatric Genomics Consortium schizophrenia study were examined. Pathways representing transcriptional regulation and gene expression, cell adhesion, apoptosis, and synapse organization showed significant association with schizophrenia. In particular, *CTCF*, *CACNB2*, and *ARL5B*, i.e. genes involved in chromatin modulation, calcium channel signaling and membrane transport, respectively, were highlighted as candidate genes for schizophrenia risk.

## Introduction

Genome-wide association studies (GWAS) have identified common susceptibility variants for numerous disorders [1], [2]. For complex diseases, however, many of the discovered variants have only a moderate or weak effect on disease risk. Due to correction for multiple testing and limited sample sizes, GWAS are likely to miss a fraction of loci with small genetic effect sizes, and researchers assume that a major fraction of heritability remains hidden for statistical reasons [3]. One way of overcoming this problem is to investigate the joint effects of multiple functionally related genes (e.g. gene-sets or pathways). Pathway-based analysis of GWAS data increases the power to detect disease related genes and, potentially, single nucleotide polymorphisms (SNPs) with small genetic effects. This approach provides valuable biological insights into the etiology of complex diseases [4].

At the time of writing, several methods are in use for the pathway-based analysis of GWAS data [5], [6], and pathway association studies have identified novel candidate genes and pathways for a range of neuropsychiatric disorders [5], [7]–[12].

Various methodological approaches to pathway association analysis are available. Maciejewski [13] has described a classification for gene-set analysis that is based upon both the statistical model used and the nature of the underlying hypothesis. This classification comprises four groups: self-contained, competitive with sample randomization, competitive with gene randomization, and parametric. The main advantages of the self-contained and the competitive with sample randomization tests are twofold. Firstly, they resemble the underlying biological experiment. Secondly, the results are amenable to statistical interpretation [14], [13].

While selection of the pathway association method is an important consideration, the power of a given pathway association study is also dependent upon other factors. These include the biological information (i.e. from gene-set and pathway databases) that is integrated into the model, the use of independent replication datasets, and the different levels of interpretation, which extend from the pathway level to the level of SNPs.

**Figure 1:**
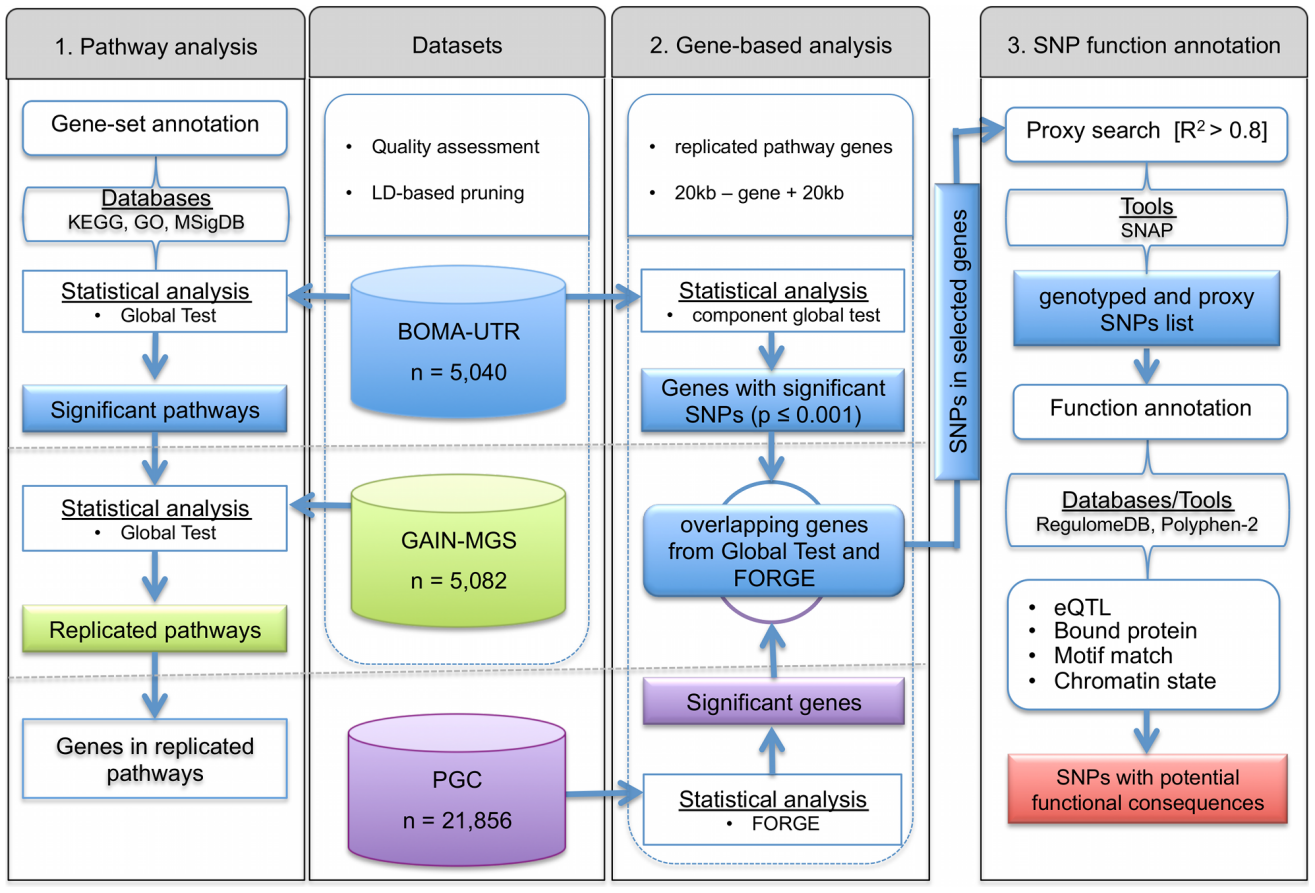
Flowchart for (1) detection and replication of schizophrenia associated pathways and (2) identification of the most informative genes, and (3) functional annotation of single nucleotide polymorphisms in the genes of interest.

As a logical consequence, researchers are now modifying analytical frameworks in order to increase their power and potential impact. To achieve this, the present study has applied a hierarchical approach (see ***Figure 1***). This approach uses three levels of evidence to unravel novel biological mechanisms with potential involvement in complex disorders. An advantage of this approach is that it builds upon previously developed and proven tools which gain synergistic effects from intersecting three different levels of evidence, i.e. evidence from the pathway-, gene-, and SNP-level. To test disease associated gene-sets and pathways, the Global Test was applied [15], [16]. To date, this well-established, self-contained pathway test has mainly been used for gene expression analyses. Subsequent identification of important risk-genes within the significant pathways was achieved using FORGE [17], while detection of the functional consequences of associated SNPs, i.e. the SNP function annotation, in the significantly associated genes was performed using RegulomeDB [18]. As part of our approach, a well-curated list of pathways and gene-set collections was integrated, and a reduction in false-positive findings was sought through the use of large-scale exploratory and independent replication samples. We applied our approach to data sets for schizophrenia (SCZ), and provide evidence for new SCZ risk genes that would otherwise have remained undetected in the investigated study samples.

## Results

### Pathway analyses

Application of the Global Test to the BOMA-UTR (MooDS SCZ consortium (BOMA)) dataset and independent data from a Dutch study (UTR), ***Table 1***) yielded 27 pathways that were significantly associated with SCZ after correction for multiple testing (False Discovery Rate (FDR)<0.05) (***Table S1A***). Of these, 14 pathways remained significant in the replication dataset. The replicated pathways are listed in ***Table 2***, together with their FDRs, nominal p-values, and SNP set sizes. The replicated pathways include the following: (i) six gene-sets from the Transcription factor Targets database (dbTFT); (ii) four Gene Ontology (GO) terms (zinc ion binding, transition metal ion binding, positive regulation of gene expression, and synapse organization); (iii) two Kyoto Encyclopedia of Genes and Genomes (KEGG) pathways (cell adhesion molecules, and apoptosis); (iv) one gene-set from the Chemical and Genomic Perturbation database (dbCGP, Kyng DNA damage by UV); and (v) one gene-set from the microRNA targets database (mir-484 targets). The gene overlap for each pathway pair is shown in ***Figure S1***. ***Table S2*** summarizes the redundancy estimates for pathways retrieved from the same source. A description and a visual depiction of pathways with similar SNP content in the BOMA-UTR dataset are provided in ***Text S1*** (section “Pathway overlap”) and ***Figure S2***, respectively. The overall gene and SNP overlap between all pairs of replicated pathways are provided in ***Table S3A*** and ***Table S3C***, respectively. For the GAIN-MGS dataset, the gene and SNP overlap information is provided in ***Table S3B*** and ***Table S3D***, respectively. The section “Subject vs SNP label permutations” in ***Text S1*** and ***Figure S3*** provides a detailed description of the results of the SNP-label permutation test coupled with the subject-sampling test.

**Table 1:** Description of individual samples.

Sample	Ancestry	Case (n)	Control (n)	Platform^a^	Reference^b^
BOMA	German	1 531	2 168	I5, I6Q, IWQ	[48], [49]
UTR	Dutch	699	642	I5	[48]
GAIN	European	1 157	1 364	A6	[50]
MGS	European	1 279	1 282	A6	[50]

a Platforms are: I5, Illumina HumanHap 550; I6Q, Illumina Human610 Quad; IWQ, Illumina Human660W-Quad; A6, Affymetrix Genome-Wide Human SNP Array 6.0.

b Publication reporting individual sample level genotypes for Schizophrenia is listed.

Discovery set: single nucleotide polymorphisms (SNPs) before pruning – 491,393; after pruning – 419,267.

Replication set: SNPs before pruning – 669,059; after pruning – 552,988.

**Table 2:** Comparisons of FDRs (BH) and p-values (P) for the BOMA-UTR and the GAIN-MGS data sets for the replicated pathways.

Description	Pathway ID	BOMA-UTR		GAIN-MGS		Number of SNPs	
		BH	P	BH	P	BOMA-UTR	GAIN-MGS
dbMIR:gagcctg,mir-484	GAGCCTG,MIR-484	1.60E-02	1.32E-04	1.01E-04	6.66E-06	1,658	2,332
dbGO:0008270:zinc ion binding*	GO:0008270	1.58E-02	8.13E-06	1.01E-04	7.46E-06	14,839	34,704
dbGO:0046914:transition metal ion binding*	GO:0046914	5.17E-04	8.20E-07	1.02E-04	1.14E-05	17,193	40,248
dbTFT::v$hnf4 q6*	V$HNF4_Q6	5.85E-04	6.42E-06	5.04E-04	9.33E-05	3,450	4,375
dbGO:0010628:positive regulation of gene expression*	GO:0010628	2.34E-02	3.21E-04	7.88E-04	1.75E-04	8,878	18,006
dbTFT:v$chop 01	V$CHOP_01	3.76E-05	1.65E-07	5.51E-03	1.63E-03	4,365	5,436
dbKEGG:04514:cell adhesion molecules (cams)	hsa04514	2.02E-02	8.45E-04	1.21E-02	4.02E-03	3,562	4,846
dbTFT:v$ciz 01*	V$CIZ_01	3.76E-05	1.63E-07	1.59E-02	5.88E-03	4,443	5,987
dbKEGG:04210:apoptosis*	hsa04210	1.97E-02	6.42E-04	3.33E-02	1.48E-02	985	1,304
dbTFT:v$sox5 01	V$SOX5_01	1.02E-03	2.01E-05	5.32E-02	2.56E-02	5,067	6,159
dbTFT:v$cebpa 01	V$CEBPA_01	4.03E-04	3.54E-06	7.84E-02	4.07E-02	4,113	5,133
dbTFT:v$ptf1beta q6	V$PTF1BETA_Q6	1.02E-03	1.60E-05	1.07E-01	6.73E-02	4,849	5,911
dbCGP:Kyng dna damage by uv	KYNG_DNA_DAMAGE_BY_UV	2.89E-02	9.89E-05	1.49E-01	9.96E-02	577	732
dbGO:0050808:synapse organization	GO:0050808	3.21E-02	2.89E-05	3.28E-01	2.92E-01	2,504	3,862

* - Significant pathways identified by more than one pathway analysis method within the BOMA-UTR data set. The test statistics obtained using the alternative algorithms are provided in Table S1B.

Note: FDR – False Discovery Rate; BH – Benjamini-Hochberg.

**Figure 2:**
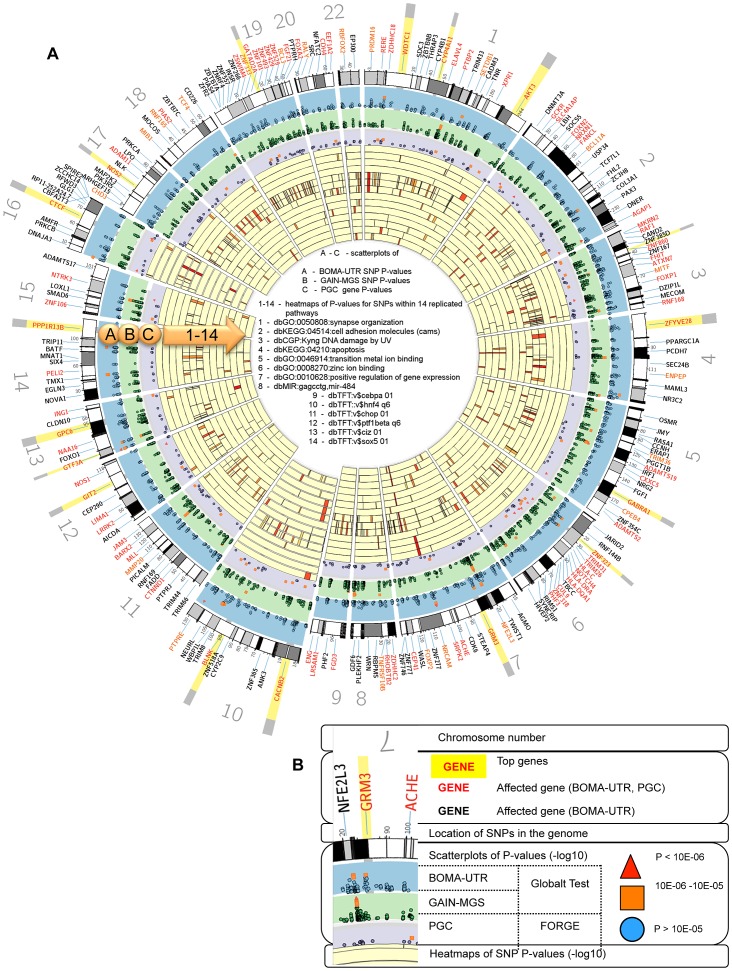
(A) Circos plots integrating the Global Test and FORGE analysis and heatmaps for the levels of single nucleotide polymorphism (SNP)- and gene significance. (B) Inset legend providing information represented by each data ring. Notes: for visibility, the implicated gene locations were zoomed in upon by up to 1200%. The inset legend image provides information represented by each ideogram. −log_10_ of the individual SNP and the gene p-values increase radially outward. The arc of each heatmap wedge maps directly to the location of the SNP in the genome. The arc width is proportional to the size of the associated gene (plus 20 kb upstream and downstream). Individual SNP p-values for the BOMA-UTR and the GAIN-MGS data sets are shown as scatterplots on ideograms A and B. The gene p-values for Psychiatric Genetics Consortium (PGC) datasets are shown as a scatterplot on ideogram C. The significance scores for genes contributing to a pathway significance are shown as heatmaps on ideograms 1–14. 1 - dbGO:0050808:synapse organization; 2 - dbKEGG:04514:cell adhesion molecules; 3 - dbCGP:Kyng dna damage by UV; 4 - dbKEGG:04210:apoptosis; 5 - dbGO:0046914:transition metal ion binding; 6 - dbGO:0008270:zinc ion binding; 7 - dbGO:0010628:positive regulation of gene expression; 8 - dbMIR:gagcctg,mir-484; 9 - dbTFT:v$cebpa 01; 10 - dbTFT::v$hnf4 q6; 11 - dbTFT:v$chop 01; 12 - dbTFT:v$ptf1bea q6; 13 - dbTFT:v$ciz 01; 14 - dbTFT:v$sox5 01. The darker the red, the higher the contribution of the SNP/gene to the association of the respective pathway. Comparing the overlapping of important genes in different pathways allows investigation of whether they lie within intersections of those pathways.

To visualize the integration of the Global Test application on a SNP-, a gene- and a pathway level, Circos plots were generated for the entire genome (***Figure 2***). These plots illustrate the impact of those individual SNPs that were annotated to the replicated pathways (whether overlapping or unique to a specific pathway) and the associated genes.

### Gene-based analysis

A total of 100 genes fulfilled the criteria described in the Methods section “Gene-based analysis with Global Test and FORGE”, i.e. these genes map to SNPs with a component Global Test p-value of <0.001 in the BOMA-UTR dataset. Of these, the following eight genes were annotated to at least four (up to eight) of the 14 replicated pathways, thus indicating their potential importance in terms of SCZ risk: *FOXP2* (eight pathways); *BCL11A* (six pathways); *PCDH7* and *RPL36P13* (five pathways respectively); and *CACNB2*, *CTCF*, *MECOM*, and *RIMS1* (four pathways respectively).

Of the genes that were annotated to the 14 replicated pathways, the top 100 were then tested in the Psychiatric Genomewide Association Study Consortium (PGC) data. Of these, significant results were obtained for 18 genes (see ***Table S4***). The vast majority of the 18 genes reside on different chromosomes, while most of the remainder reside on different chromosome arms. It therefore seems reasonable to assume that they represent independent signals, which results in a p-value of 0.004 for an enrichment of SCZ-associated genes among the 100 top genes. Included in the list of 18 replicated genes are known SCZ susceptibility genes, such as *NRXN1*, *GRM3*, and *MMP16*. Two of the eight most frequent genes in the top 14 pathways were also among the nominally significant genes in the gene-based FORGE analysis, i.e. *CACNB2* (p = 8.57×10^−4^) and *CTCF* (p = 0.015). Given the overlap (approx. 1,200 cases) between the PGC sample (FORGE analyses) and the present discovery sample (component Global Test), we opted to analyze the PGC dataset without including our discovery dataset. These analyses generated results of the same order of magnitude for both genes (*CACNB2*: p = 0.0090; *CTCF*: p = 0.0320). While *CACNB2* showed a trend towards association in an independent dataset from Denmark (p = 0.0970), thus supporting the strong signal from the PGC data, *CTCF* was found to be strongly associated in the same independent Danish sample (p = 0.0075).

### Potential functional consequences of SNPs in CTCF

**Figure 3:**
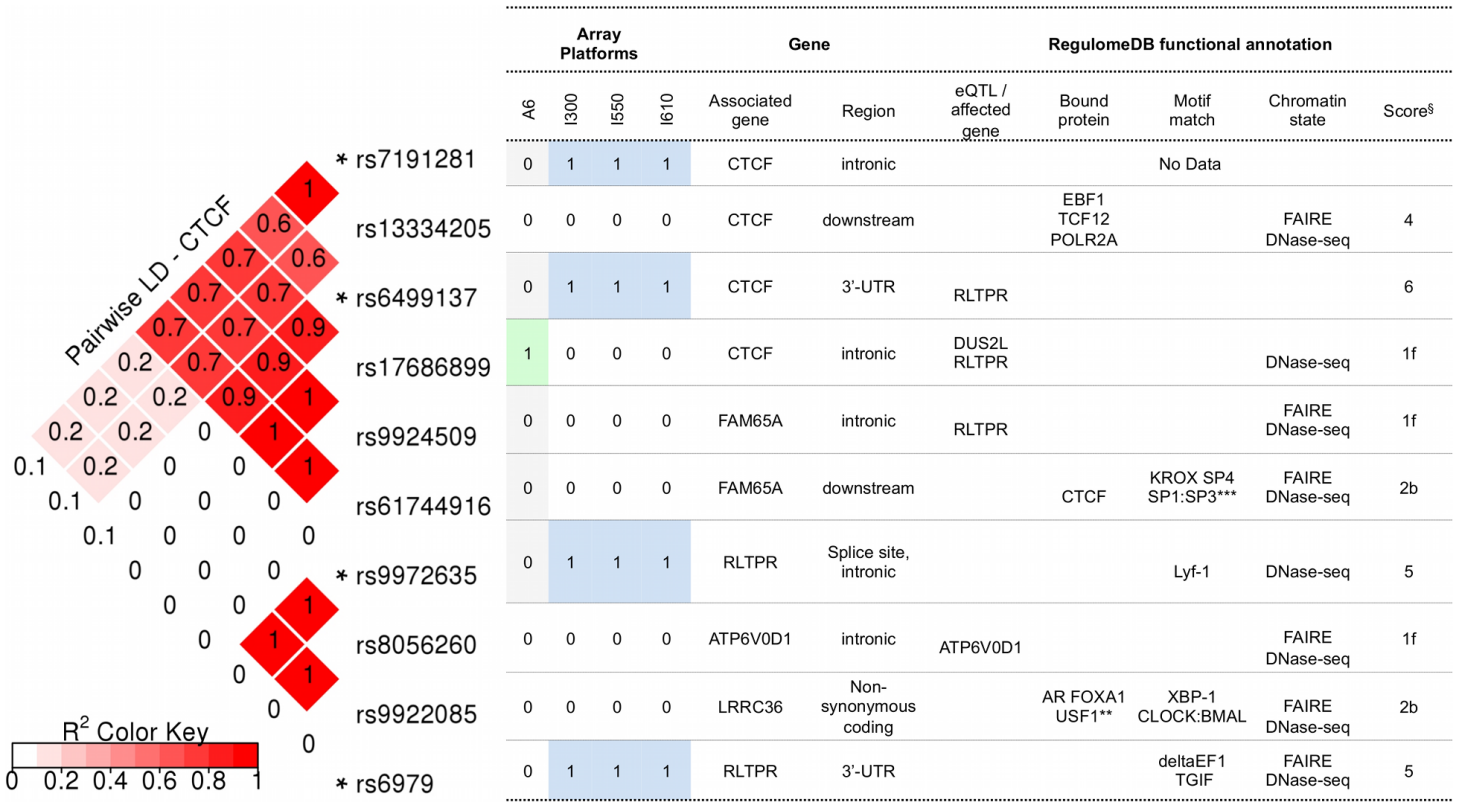
RegulomeDB functional annotation for SNPs in CTCF and its regulatory regions. Notes: * genotyped in the BOMA-UTR data set and sorted by their genomic coordinates. SNPs are within or 20 kb upstream and downstream of *CTCF*. ** *AR FOXA1 USF1 CDX2 HNF4A TRIM28 USF2 TCF4 HDAC2 SP1 BHLHE40*. *** *KROX SP4 SP1:SP3 HIC1 Zif268 Sp4 Sp1 SP1 Egr*. § RegulomeDB score: [1f] - likely to affect binding and linked to expression of a gene target; [2b] - likely to affect binding; [4], [5], [6] - minimum binding evidence.

Polyphen-2 predicted that the coding SNPs of interest in *CTCF* were “benign”, whereas SIFT predicted that they were “tolerated” (***Table S5***). ***Figure 3*** illustrates the potential consequences predicted for SNPs in *CTCF* and its regulatory regions. These include SNPs genotyped in the present discovery study and SNPs identified as their proxies using SNAP. For the latter, only those that were annotated by RegulomeDB as being (1) likely to affect DNA binding of the protein and linked to expression of a gene target, or (2) likely to affect DNA binding, are listed. The complete functional annotation data for the SNPs of *CTCF* are provided in ***Table S5***. All genotyped SNPs annotated to *CTCF* showed a significant (component Global Test p-value of ≤0.05) contribution to pathway associations. Of these, rs6499137 and rs7191281 were located at the 3′-UTR and the intron of *CTCF*, respectively. Given the 20 kb flanking region allowed for assigning the SNPs to a gene, the other two SNPs were considered to be shared with the neighboring gene *RLTPR*. Based on the functional annotation with the RegulomeDB database, the 3′-UTR SNP of *CTCF* (rs6499137) and its proxies were considered to be associated with the altered expression of the neighboring gene *RLTPR* (***Figure 3***, ***Table S5***). One of the proxies (rs17686899) overlaps with a number of functional elements, such as open chromatin region, the binding sites for different transcription factors, and regions with certain histone modifications across many cell types. This suggests that the SNP was likely to affect the binding of a number of transcription factors to the genomic region of this gene. The respective expression quantitative trait loci (eQTL) information suggested that the SNP was likely to affect the expression of two genes, i.e. *DUS2L* and *RLTPR*. Among the *CTCF*-annotated SNPs, the intronic SNP of *CTCF*, rs7191281, was one of the top SNPs (component Global Test p-value of <0.001) contributing to the association of *CTCF* (and the association of the four replicated pathways containing *CTCF*). In addition, this SNP had the lowest p-value in the analyses of the PGC SCZ sample. While no information concerning functionality was available in the RegulomeDB database for this intronic SNP of *CTCF*, its proxy, rs13334205, was annotated with strong functional consequences. This proxy SNP was located in the regulatory region of *CTCF* and overlapped with the binding site of DNA-binding proteins, such as *EBF1*, *TCF12*, *POLR2A*, in an open chromatin region (***Figure 3***, ***Table S5***).

### Potential functional consequences of SNPs in CACNB2

The complete functional annotation data for the SNPs of *CACNB2* are provided in ***Table S6***. The positions of the majority of the genotyped and the proxy SNPs of *CACNB2* overlapped a motif match to the FOX (*FOXP1*, *FOXJ1*, *FOXJ2*) and GATA (*GATA1*, *GATA3*) family motifs in open chromatin regions. Among the SNPs mapped to *CACNB2*, rs12257556 and its proxy rs4748474 were annotated with the strongest functional consequences. These intronic SNPs were eQTLs for *ARL5B*, and overlapped an open chromatin region. The proxy SNPs rs35803482 and rs7897710 both overlap with the binding sites of *RAD21*, *SMC3*, *CTCF*, and have a motif match for *FOXP1*. The intronic SNP rs2799573 (which was also the most highly associated SNP of CACNB2 in the PGC data) lies in the binding region of a number of proteins, such as *CDX2*, *CTCF*, *JUN*, *JUND*, *MEF2A*, *RAD21*, and *SMC3* (***Table S6***), as identified in the ENCODE ChIP-seq data across a diverse set of cell types.

## Discussion

### SCZ GWAS data analyses

In the present study, a genome-wide pathway association analysis was performed by means of the Global Test. The analyses involved well-curated descriptions of 7,350 pathways, and were carried out on large-scale discovery and replication datasets. A gene-based analysis of genes with a high contribution to the significance of the top pathways was then performed using the SCZ GWAS results of the PGC. Finally, a functional SNP-based analysis of the top hit genomic regions was conducted. Through this hierarchical approach, we were able to replicate pathway findings from previous studies of SCZ and detect novel pathways and genomic regions with an association to SCZ in the investigated samples. In the discovery set, we detected evidence for a significant contribution of 27 pathways. Of these, 14 remained significant in the replication dataset. The 14 replicated pathways are involved in transcriptional regulation and gene expression, synapse organization, cell adhesion, and apoptosis.

Previous pathway analyses of SCZ GWAS data have identified associations with pathways that are mainly involved in processes critical to synaptic function, neurodevelopment, cell adhesion, the immune system, the estrogen biosynthetic process, and apoptosis [10], [19], [20]. One of the 14 significant pathways in the present study, i.e. cell adhesion, was also the most significant pathway in the study by O'Dushlaine et al. [10]. Jia et al. [19] reported nominal significance for the following four pathways: CARM_ER (CARM1 and Regulation of the Estrogen Receptor); glutamate metabolism; TNFR1; and TGF beta signaling. Glutamate is implicated in synaptic neurotransmission, and TGF-beta and TNFR1 signaling are involved in several cellular processes, including apoptosis and excitotoxicity. The top hit pathways “synaptic organization” and “apoptosis” from the present study are thus consistent with the results of Jia et al [19].

However, the majority of pathways with significant association to SCZ in the present study are novel, and they are mainly involved in transcriptional regulation and gene expression. One reason for the failure of previous pathway-based studies of SCZ to generate similar findings may have been that they focused mainly on gene sets from the KEGG and BioCarta databases, whereas we accessed several pathway databases. These included the GO database, as well as special gene-set collections on chemical and genomic perturbations (dbCGP), and transcriptional regulation such as dbTFT and dbMIR. It should be noted that only few of our 14 replicated pathways achieved significance in the analysis of our discovery sample using GRASS [21], gseaSNP [22], and ALIGATOR [23]; see ***Text S1*** and ***Table S1C***). The difference in results can be explained by the different assumptions these alternative pathway approaches rest on.

As part of our hierarchical approach, we aimed to identify which genes in a particular pathway could be responsible for the association with SCZ risk. Integration of gene-based analysis facilitated both the prioritization of potential candidate genes and more precise formulation of hypotheses concerning the functional consequences of the potential pathway perturbations (i.e. at the gene- and SNP-level). In particular, we explored how variants that emerged as being of importance for our pathway- and gene-based signals might affect the function and regulation of other genes.

In the gene-based analysis, *CACNB2* and *CTCF* showed the strongest evidence for association with SCZ in both the present samples and in those of the PGC. The gene *CACNB2* encodes an auxiliary voltage-dependent L-type calcium-channel subunit that is mainly expressed in heart and brain tissue [24]. This subunit is essential for normal surface expression, adequate trafficking, and functioning of voltage-gated calcium channels [24]. Recently, *CACNB2* was among four loci with genome-wide significance in a cross-disorder analysis of GWAS data for autism spectrum disorder, attention deficit-hyperactivity disorder, bipolar disorder, major depressive disorder, and SCZ [25]. Previously, *CACNB2* had been one of the top hit regions in a GWAS of bipolar disorder I in a Han Chinese population [26]. Functionally, the calcium channel beta-2 subunit encoded by *CACNB2*, together with the calcium channel alpha(2)/delta subunit, affects the kinetics and expression of Ca(V)1.2 (encoded by *CACNA1C*) [27]. *CACNA1C* is a well-established susceptibility gene for bipolar disorder, SCZ, and major depressive disorder [25], [28]–[31]. The RegulomeDB search of genotyped SNPs and their proxies in *CACNB2* resulted in the detection of the intronic SNPs rs12257556 and rs10764566, and these were eQTLs for *ARL5B*. The gene *ARL5B* encodes a *trans*-Golgi network localized small G protein that has been described as a key regulator of retrograde membrane transport [32]. Altered *ARL5B* expression may be involved in the dysregulation of axonal transport. Interestingly, a previous study found that the transcript of one of the most widely studied susceptibility genes for SCZ, *DISC1*, was an interacting molecule for a motor protein of axonal transport [33]. It is of note that SNPs (both genotyped and proxies) at the *CACNB2* locus suggested an interplay with our second gene of interest, i.e. *CTCF*. Such a connection is also suggested with *RAD21*. A substantial body of literature describes an interaction between *RAD21* and *CTCF*, particularly in neurons [34], [35]. Although few data are available on a potential interaction between *CACNB2* and *RAD21*/*CTCF*, moderate evidence is available from several protein-protein interaction databases (data not shown) for an interplay between *CTCF*, *RAD21*, and *ARL5B*.

*CTCF* encodes a transcriptional regulator protein with 11 conserved zinc finger domains, and is an important modulator of conformational changes in chromatin [36]. A recent study of conditional knockout of the *ctcf* gene in mice demonstrated that CTCF was a key regulator of neuronal differentiation, and was essential for neuronal diversity and functional neural networks [37]. The authors showed that CTCF was required for appropriate dendritic arborization and synapse formation, since it controlled clustered protocadherin expression. Previous studies have shown an association between genetic variation in the protocadherin gene cluster and SCZ [38], [39]. Our result adds to this body of research the finding that transcriptional regulation of genes essential for neuronal diversity, such as the regulation of protocadherins by *CTCF*, may alter synaptic connectivity and thus contribute to the etiology of SCZ. Intriguingly, evidence from the majority of *CTCF* SNPs (both genotyped and proxies) suggested that the variants influence *RLTPR* expression (***Figure 3***). The *RLTPR* gene is expressed in several brain regions (EMBL-EBI Expression Atlas; http://www.ebi.ac.uk/gxa/gene/ENSG00000159753). The resulting protein has a RGD (Arginine-Glycine-Aspartic acid) motif [40]. This is a universal cell recognition site of extracellular proteins and interacts with a family of cell-surface receptors, such as integrins for cell-adhesion molecules [41]. Together with the replicated KEGG pathway cell adhesion molecules, this finding strongly supports the hypothesis that modulation of adhesion, and interactions between cells as well as cell and the extracellular matrix, are implicated in the etiology of SCZ.

Another top hit gene in the present study was *FOXP2*, which was among the top genes in eight of the 14 most implicated pathways. FOXP2 (forkhead-box P2) is a transcription factor with an essential role in the development of speech and language regions in the brain. The fact that SCZ patients often show language impairments such as reading difficulties [42] renders *FOXP2* a plausible SCZ candidate gene. Interestingly, a previous study reported an association between genetic variation in *FOXP2* and SCZ in a Han Chinese population [43]. Furthermore, Walker et al. [44] identified *FOXP2* as an inhibitor of the promoter activity and protein expression of *DISC1*. The present study supports the hypothesis that *FOXP2* plays an important role in SCZ on the level of the transcriptional regulation of target genes.

The association with the apoptosis pathway was driven predominantly by a SNP which mapped to *AKT3*. Besides being detected via the Global Test, this gene was the most significantly associated gene in the FORGE analysis of the PGC data. AKT3 is a serin/threonine protein kinase, and is a member of the AKT family. It is involved in many biological processes, including apoptosis and cellular proliferation [45]. In a recent study by Diez et al. [46], AKT3 was identified as a modulator of the fine regulation of apoptotic processes and axon growth. Disruption of AKT3 significantly reduced axon length and viability of neurons in cell culture [46]. Moreover, AKT3 is the most abundant AKT member in the brain during neurogenesis. AKT3 controls brain size, and research has shown that genetic variation (duplication and point mutation) of *AKT3* contributes to hemimegalencephaly [47].

In conclusion, the present study demonstrated that use of information from databases focusing on cell-regulatory networks together with information from traditional pathway database resources can facilitate the identification of susceptibility factors for the complex neuropsychiatric disease SCZ. Through the application of a well-designed hierarchical framework, our study highlighted the importance of calcium channel signaling, cell adhesion, and the modulation of transcriptional regulation implicated in neuronal diversity, neurite growth, and synapse formation in the etiology of SCZ. In particular, *CTCF* and *CACNB2* (and possibly *ARL5B*) were identified as SCZ candidate genes.

## Materials and Methods

### Ethics statement

Each participant provided written informed consent prior to inclusion and all aspects of the study complied with the Declaration of Helsinki. The study was approved by the ethics committees of all study centers. For the German samples, this comprised the Ethics Committee of the Rheinische Friedrich-Wilhelms-University Medical School in Bonn, Ethics Committee “Medizinische Ethik-Kommission II” of the University of Heidelberg, the Ethics Committee of the Friedrich-Schiller-University Medical School in Jena, and the Ethics Committee of the Ludwig-Maximilians-University Munich. Samples obtained through dbGaP were collected using institutional review board-approved protocols in three studies, i.e. Schizophrenia Genetics Initiative (SGI), Molecular Genetics of Schizophrenia Part 1 (MGS1), and MGS2.

### Data sets

Participants from four datasets were included (***Table 1***). The discovery set was the BOMA-UTR sample. This consisted of data from the MooDS SCZ consortium (BOMA) [48], [49], and independent data from a Dutch study (UTR) [48], and comprised 2,230 SCZ cases and 2,810 controls. The replication set consisted of the GAIN [dbGaP accession number: phs000021.v2.p1], and the MGS [dbGaP accession number: phs000167.v1.p1] datasets, and comprised 2,436 SCZ cases and 2,646 controls [50]. The BOMA and MGS samples were also used in the PGC SCZ study. An overlap of 80% existed between the PGC study and the sample used in the present pathway-based analysis.

### Linkage disequilibrium (LD)-based SNP pruning

To accommodate the Global Test's assumption of independence between variables, the SNP set was reduced according to a variance inflation factor (VIF) and using a sliding window approach, as implemented in PLINK [51] (http://pngu.mgh.harvard.edu/purcell/plink/, version 1.07). A VIF of 100 was used. The window size was set at 50 SNPs, and was shifted by 5 SNPs at each step. An LD-based pruned set of SNPs (***Table 1***) was then considered for mapping to pathways. A detailed description of this procedure is provided in ***Text S1*** (section “SNP independence and LD-based SNP pruning”) and in ***Table S7***.

### PGC sample

For the gene-based analysis, PGC data (https://pgc.unc.edu/ResultFiles/pgc.scz.2012-04.zip) were used.

### Annotation of SNPs to genes

SNPs were annotated with information from dbSNP Build 127. The “seq-gene” file containing information for annotating the SNP rs numbers to ENTREZ gene IDs was downloaded from the NCBI ftp website (BUILD 36.3). SNPs were assigned to a gene if the SNP was located within the genomic sequence or within 20 kb of the 5′ and 3′ ends of the first and last exons in order to account for important regulatory regions [52]. If a SNP was within a region shared by more than one gene, it was assigned to all genes (for details see ***Text S1***).

### Pathway and gene-set databases

Selected gene-set collections were accessed from the Molecular Signatures Database (MSigDB, version 3.0) [53] website (http://www.broadinstitute.org/gsea/msigdb). This included the pathways from BioCarta (217 pathways), Chemical and Genomic Perturbations (1,825 gene-sets), Reactome (775 pathways), MicroRNA Targets (176 gene-sets), and Transcription Factor Targets (456 gene-sets). Information concerning GO terms [54] and KEGG pathways [55], [56] was obtained from the respective R packages (3,686 GO terms; GO.db, version 2.5.0; 215 KEGG pathways; R package KEGG.db, version 2.5.0). At the time of data retrieval (June, 2011), these repositories were more up-to-date than the MSigDB database. A total of 7,350 pathways were included. These were represented by 237 788 (53.7%) of the SNPs in the BOMA-UTR dataset. Hence 53.7% of SNPs genotyped in the exploration samples were mapped to pathways. For the SNP data, SNP effect was coded as an allele dose effect (0, 1, 2). Detailed information on the pathway information overlap and redundancy is provided in ***Text S1*** (section “Choice of pathways and gene-sets”) and in ***Table S2*** and ***Figure S1***.

### Pathway analysis with the Global Test

For the pathway-based analysis, the Global Test [15] was used (R package *globaltest*, version 5.12.0; ***Figure 1***). The Global Test takes the individual level GWAS data as an input, and tests whether the global polymorphism pattern of a group of genes is significantly associated with the phenotype of interest. To account for both a potential underlying correlation structure and pathway and/or gene size, the Global Test with subject sampling was applied on the basis of 10,000 permutations of case-control status [15]. To study the impact of pathway and/or gene size in more detail, a SNP label permutation test was performed (for detailed information see ***Text S1***, section “Subject vs SNP label permutations”).

At the discovery stage of the analysis, less conservative correction for multiple testing was applied in order to prioritize the identification of associated pathways. This was a legitimate approach, since any false positives would be controlled for in the replication analysis. Multiplicity correction was applied for each individual collection of pathways/gene-sets. For pathways/gene-sets retrieved from the KEGG, Reactome, and MSigDB gene-set collections, the pathway scores were corrected for multiple testing using the Benjamini-Hochberg method [57]. A pathway was considered to be significantly associated with the phenotype of interest (i.e. SCZ) if the false discovery rates from all three of the following were <0.05: (i) un-permuted test; (ii) the subject-sampling test; and (iii) the SNP-label permutation tests. The resulting list of significant pathways was ranked according to the false discovery rate obtained from the SNP-label permutation tests. For the GO terms, correction for multiple testing was performed using the Focus Level method [58]. A GO term was considered to be significant if both of the following were <0.05: (i) the focus level obtained from the un-permuted test; and (ii) the false discovery rate obtained from the subject-sampling test. To account for a gender-specific variance in the perturbed pathways, control for gender was used as a covariate [15].

### Component Global Test

To estimate the contributions of individual SNPs to a pathway- or a gene association, the component global test was performed using the *covariates* function implemented in the R package *globaltest* [15]. Throughout the text, the single SNP p-values obtained using the Global Test refer to the results obtained using the component global test.

### The Global Test with the replication dataset

Only pathways that were significantly associated with SCZ in the discovery set were followed-up (***Figure 1***, step 1). All tests in the follow-up step were performed as described above, with the exception that all tested pathways were subjected to Benjamini-Hochberg correction for multiple testing. Possible stratification in the data was investigated using a multi-dimensional scaling (MDS) approach. MDS covariates were obtained from PLINK using a previously described protocol [48]. To correct for the potential effect of stratification on the association test, the Global Test was run with four leading MDS dimensions as covariates.

### Gene-based analysis with Global Test and FORGE

The aim of the second step (***Figure 1***, gene-based analysis) was to identify genes of particular importance to the replicated pathways. Genes that mapped to one or more of the identified pathways were analyzed (***Figure 1***, step 2). First, the component global test was performed for every individual SNP that was annotated to the replicated pathways. SNPs with a component global test p-value of <0.001 in the BOMA-UTR dataset were then annotated to genes. These genes are referred to as “top genes” in the subsequent text. Gene-based analysis of PGC data for the top genes was then conducted using FORGE [17] As with the Global Test, the analyses focused on genomic sequences that included both the genes themselves and a 20 kb window on either side of the respective gene to account for important regulatory regions. Along with the summary statistics of the PGC, genotype data from the European HapMap 3 samples were used (CEU and TSI). Details of the program and the test statistic used to calculate the gene-based p-values (fixed-effects Z score method) are provided elsewhere [17]. Genes that remained nominally significant (p<0.05) in both the component global test and the FORGE analyses were considered for the third step of the analyses (SNP function annotation). No correction for multiple testing was performed. However, replication of our most interesting findings was sought in an independent dataset from Denmark. Detailed information on these Danish samples is provided elsewhere [59].

### SNP function annotation

The third step (***Figure 1***, SNP function annotation) focused on genes identified in step 2. Evidence that SNPs annotated to these genes are implicated in SCZ was sought by investigating the potential consequences of SNPs in terms of gene regulation or function. For each gene of interest, we first selected all SNPs that were annotated to this gene and which had shown evidence for association with SCZ in the discovery dataset (Global Test, p≤0.05). To account for the relevant information from other correlated SNPs, we then identified all SNPs from the 1000 genomes project (pilot project) [60] that showed strong LD with the associated SNPs (r^2^>0.8, maximum distance between both SNPs = 500 kb). The webtool SNAP [61] (Version 2.2) was used. Each query SNP was included as its own proxy. RegulomeDB [18] and Polyphen-2/SIFT [62], [63] were used for the functional classification of non-coding and coding SNPs, respectively.

## Supporting Information

Figure S1 The heatmap of the level of gene overlap between the 27 schizophrenia associated pathways. The values in the cells indicate the maximum fraction overlap of the genes in a pathway (listed on y-axis). The corresponding pathway name in the x-axis is a pathway with the highest overlap (self-overlap is excluded).
(TIF)

Figure S2 Hierarchical clustering of replicated pathways. The data are the counts of overlapping implicated single nucleotide polymorphisms, as detected using the Global Test in the BOMA-UTR dataset.
(TIF)

Figure S3 Comparison of the p-values obtained from the single nucleotide polymorphism-label permutation and subject-sampling test for all gene-sets.
(TIF)

Table S1 Comparisons of FDRs (BH) and P-values (P) for (**A**) BOMA-UTR datasets for top 27 schizophrenia associated pathways identified by the GlobalTest performed to account for gender differences, linkage disequilibrium-structure, and gene-set size, (**B**) for the independent datasets (BOMA, UTR, GAIN, and MSG) for the top 27 schizophrenia associated pathways, (**C**) for BOMA-UTR dataset for top 14 replicated schizophrenia associated pathways identified by various analysis methods.
(DOC)

Table S2 Comparison of redundancies in the subsets of the 6 pathway databases/gene-set collections.
(DOC)

Table S3 (**A**) Genes overlapping between the 14 replicated pathways in the BOMA-UTR dataset **and** (**B**) the GAIN-MGS dataset. (**C**) Single nucleotide polymorphisms overlapping between the 14 replicated pathways in the BOMA-UTR dataset and (**D**) the GAIN-MGS dataset.
(DOC)

Table S4 List of schizophrenia (SCZ) associated genes, their p-values (FORGE analysis), and membership in the SCZ associated pathways discovered and replicated in the present study. Pathways in bold also showed an overall association using one of the other three methods (ALIGATOR, GRASS, gseaSNP) applied in the present study.
(DOC)

Table S5 Potential functional consequences of CTCF associated SNPs.
(XLS)

Table S6 Potential functional consequences of CACNB2 associated SNPs.
(XLS)

Table S7 The Global Test results for the discovered gene-sets remained significant when the test was repeated with varying degrees of multicollinearity in the data.
(DOC)

Text S1 Description of supplementary results and methods.
(DOC)
