# Supplementary figures and images for: Integrated Pathway-Based Approach Identifies Association between Genomic Regions at CTCF and CACNB2 and Schizophrenia
Source: PLoS Genet. 2014 Jun 5;10(6):e1004345. doi: 10.1371/journal.pgen.1004345 (PMC4046913; doi:10.1371/journal.pgen.1004345)

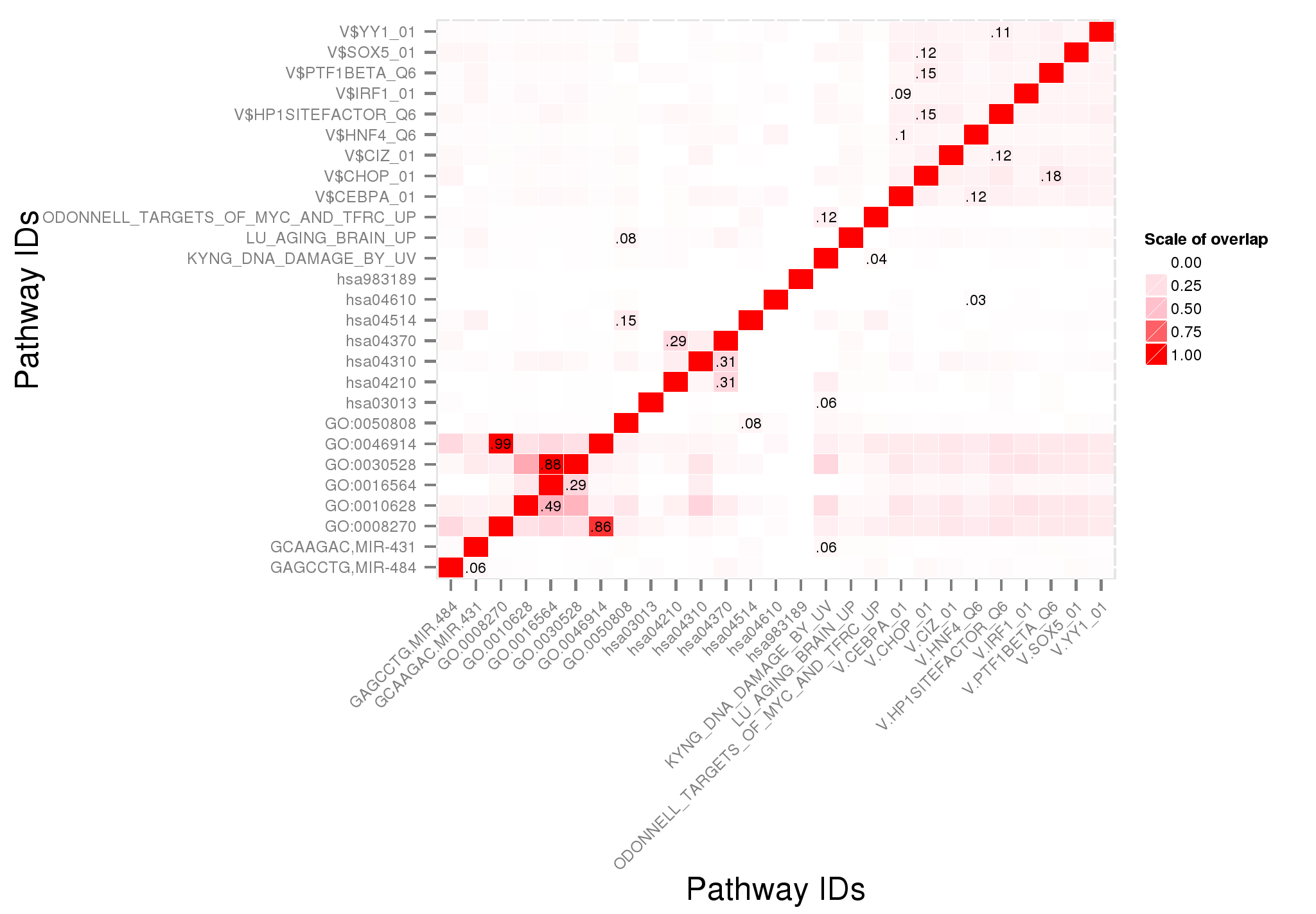

Supplement: Figure S1 — The heatmap of the level of gene overlap between the 27 schizophrenia associated pathways. The values in the cells indicate the maximum fraction overlap of the genes in a pathway (listed on y-axis). The corresponding pathway name in the x-axis is a pathway with the highest overlap (self-overlap is excluded). (TIF) [file pgen.1004345.s001.tif]

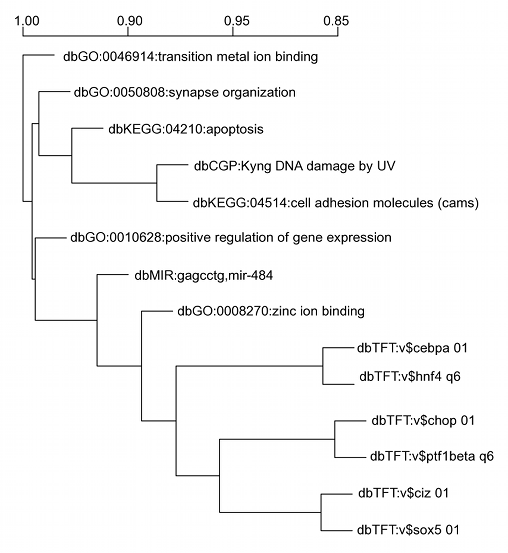

Supplement: Figure S2 — Hierarchical clustering of replicated pathways. The data are the counts of overlapping implicated single nucleotide polymorphisms, as detected using the Global Test in the BOMA-UTR dataset. (TIF) [file pgen.1004345.s002.tif]

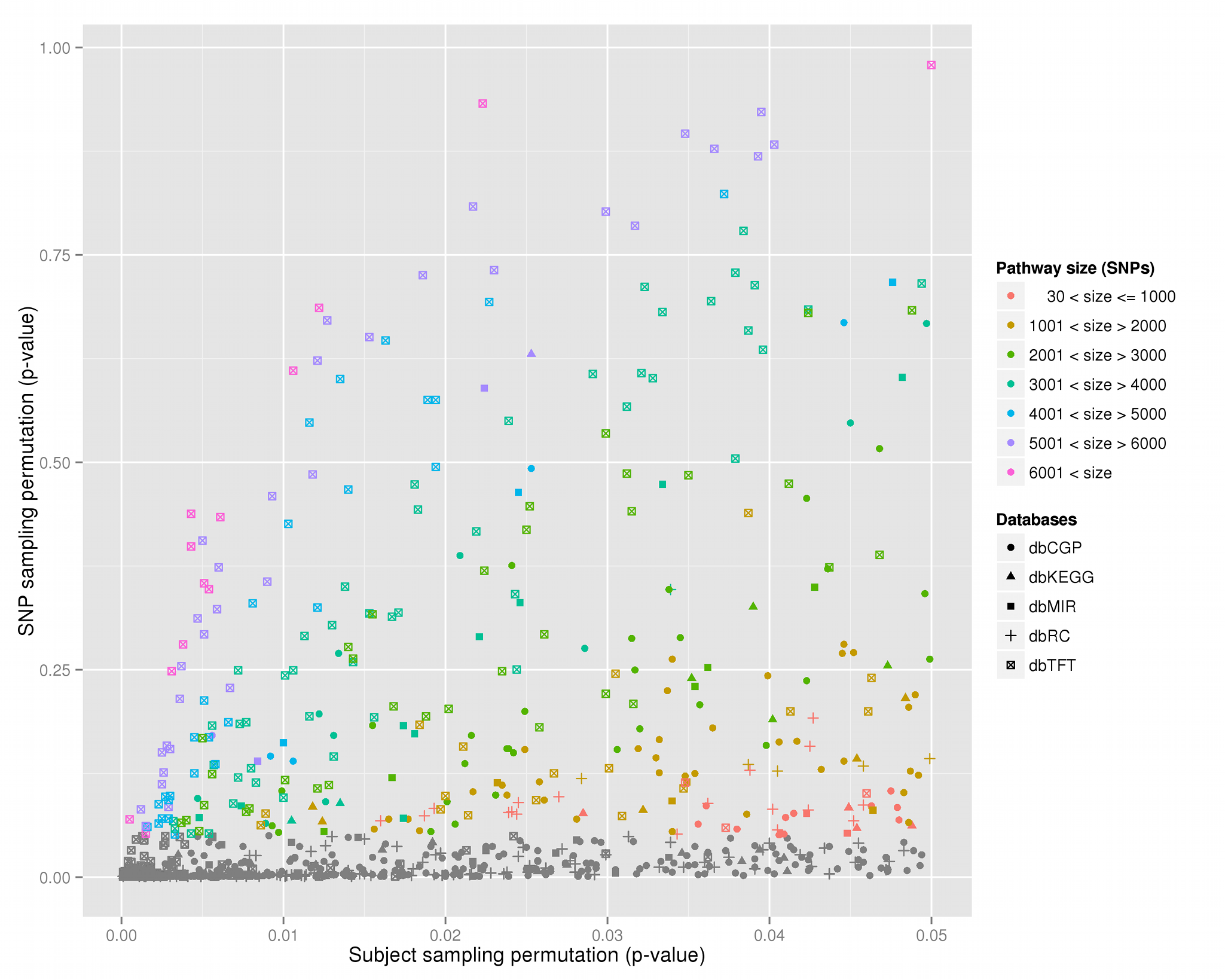

Supplement: Figure S3 — Comparison of the p-values obtained from the single nucleotide polymorphism-label permutation and subject-sampling test for all gene-sets. (TIF) [file pgen.1004345.s003.tif]
