# Supplementary material for: Integrated Pathway-Based Approach Identifies Association between Genomic Regions at CTCF and CACNB2 and Schizophrenia
Source: PLoS Genet. 2014 Jun 5;10(6):e1004345. doi: 10.1371/journal.pgen.1004345 (PMC4046913; doi:10.1371/journal.pgen.1004345)
Supplement: Table S1 — Comparisons of FDRs (BH) and P-values (P) for (A) BOMA-UTR datasets for top 27 schizophrenia associated pathways identified by the GlobalTest performed to account for gender differences, linkage disequilibrium-structure, and gene-set size, (B) for the independent datasets (BOMA, UTR, GAIN, and MSG) for the top 27 schizophrenia associated pathways, (C) for BOMA-UTR dataset for top 14 replicated schizophrenia associated pathways identified by various analysis methods. (DOC) [file pgen.1004345.s004.doc]

**Table S1A** Comparisons of FDRs (BH) and P-values (P) for BOMA-UTR datasets for top 27 SCZ associated pathways identified by the GlobalTest performed to account for the gender differences, LD-structure and gene-set size.

| **Description** | **Global Test default** | | **Global Test with gender as covariate** | | **Subject sampling test** | | **SNP-label permutation** | |
| --- | --- | --- | --- | --- | --- | --- | --- | --- |
|  | **BH** | **P** | **BH** | **P** | **BH** | **P** | **BH** | **P** |
| dbMIR:gagcctg,mir-484 | 1.60E-02 | 1.32E-04 | 3.31E-02 | 2.22E-04 | 1.17E-02 | 2.00E-04 | 9.25E-03 | 9.99E-04 |
| dbMIR:gcaagac,mir-431 | 1.60E-02 | 3.86E-04 | 3.31E-02 | 4.35E-04 | 1.17E-02 | 2.00E-04 | 9.25E-03 | 9.99E-04 |
| dbGO:0008270:zinc ion binding | 1.58E-02 | 8.13E-06 | 2.85E-04 | 1.90E-04 | 4.50E-04 | 1.00E-04 | 2.13E-01 | 1.78E-01 |
| dbGO:0010628:positive regulation of gene expression | 2.34E-02 | 3.21E-04 | 4.90E-03 | 4.90E-03 | 2.00E-04 | 2.50E-03 | 5.94E-01 | 5.94E-01 |
| dbGO:0016564:transcription repressor activity | 1.55E-02 | 2.46E-05 | 8.80E-05 | 4.04E-05 | 2.00E-04 | 1.00E-04 | 3.00E-03 | 9.99E-04 |
| dbGO:0030528:transcription regulator activity | 1.55E-02 | 2.94E-05 | 8.80E-05 | 4.40E-05 | 2.00E-04 | 3.00E-04 | 5.99E-03 | 3.00E-03 |
| dbGO:0046914:transition metal ion binding | 5.17E-04 | 8.20E-07 | 8.80E-05 | 3.49E-05 | 2.00E-04 | 1.00E-04 | 1.23E-01 | 8.19E-02 |
| dbGO:0050808:synapse organization | 2.34E-02 | 2.89E-05 | 7.91E-04 | 6.60E-04 | 6.00E-04 | 5.00E-04 | 3.00E-03 | 9.99E-04 |
| dbKEGG:03013:rna transport | 3.20E-02 | 3.26E-03 | 1.65E-02 | 5.40E-04 | 3.31E-02 | 2.90E-03 | 3.46E-02 | 5.00E-03 |
| dbKEGG:04210:apoptosis | 1.97E-02 | 6.42E-04 | 1.65E-02 | 3.93E-04 | 1.84E-02 | 6.00E-04 | 9.34E-03 | 9.99E-04 |
| dbKEGG:04310:wnt signaling pathway | 8.32E-03 | 1.55E-04 | 1.65E-02 | 7.14E-04 | 1.61E-02 | 3.00E-04 | 9.34E-03 | 9.99E-04 |
| dbKEGG:04370:vegf signaling pathway | 7.23E-03 | 4.36E-05 | 1.50E-02 | 8.14E-05 | 1.43E-02 | 1.00E-04 | 9.34E-03 | 9.99E-04 |
| dbKEGG:04514:cell adhesion molecules (cams) | 2.02E-02 | 8.45E-04 | 1.87E-02 | 1.13E-03 | 1.84E-02 | 6.00E-04 | 9.34E-03 | 9.99E-04 |
| dbKEGG:04610:complement and coagulation cascades | 2.16E-02 | 1.40E-03 | 4.42E-02 | 3.99E-03 | 2.55E-02 | 1.90E-03 | 9.34E-03 | 9.99E-04 |
| dbRC:983189:kinesins | 5.82E-02 | 1.06E-04 | 4.87E-02 | 8.83E-05 | 5.51E-02 | 1.00E-04 | 2.62E-02 | 9.99E-04 |
| dbCGP:Kyng dna damage by uv | 2.89E-02 | 9.89E-05 | 6.18E-02 | 2.86E-04 | 3.42E-02 | 3.00E-04 | 2.00E-02 | 9.99E-04 |
| dbCGP:Lu aging brain up | 3.17E-02 | 2.95E-04 | 7.46E-02 | 6.73E-04 | 4.15E-02 | 5.00E-04 | 2.00E-02 | 9.99E-04 |
| dbCGP:Odonnell targets of myc and tfrc up | 4.70E-02 | 7.38E-04 | 7.46E-02 | 8.64E-04 | 4.73E-02 | 6.00E-04 | 2.00E-02 | 9.99E-04 |
| dbTFT:v$cebpa 01 | 4.03E-04 | 3.54E-06 | 4.51E-04 | 3.96E-06 | 2.17E-03 | 1.00E-04 | 7.54E-03 | 1.01E-03 |
| dbTFT:v$chop 01 | 3.76E-05 | 1.65E-07 | 2.18E-04 | 9.56E-07 | 2.17E-03 | 1.00E-04 | 7.54E-03 | 1.01E-03 |
| dbTFT:v$ciz 01 | 3.76E-05 | 1.63E-07 | 4.96E-05 | 1.09E-07 | 2.17E-03 | 1.00E-04 | 7.54E-03 | 1.01E-03 |
| dbTFT::v$hnf4 q6 | 5.85E-04 | 6.42E-06 | 4.03E-04 | 2.65E-06 | 2.17E-03 | 1.00E-04 | 7.54E-03 | 1.01E-03 |
| dbTFT:v$hp1sitefactor q6 | 1.18E-03 | 3.63E-05 | 2.82E-03 | 6.81E-05 | 2.17E-03 | 1.00E-04 | 7.54E-03 | 1.01E-03 |
| dbTFT:v$irf1 01 | 4.03E-04 | 2.66E-06 | 1.54E-03 | 1.69E-05 | 2.17E-03 | 1.00E-04 | 7.54E-03 | 1.01E-03 |
| dbTFT:v$ptf1beta q6 | 1.02E-03 | 1.60E-05 | 2.53E-03 | 5.55E-05 | 2.17E-03 | 1.00E-04 | 7.54E-03 | 1.01E-03 |
| dbTFT:v$sox5 01 | 1.02E-03 | 2.01E-05 | 2.08E-03 | 2.74E-05 | 2.17E-03 | 1.00E-04 | 7.54E-03 | 1.01E-03 |
| dbTFT:v$yy1 01 | 1.25E-03 | 5.22E-05 | 2.53E-03 | 5.32E-05 | 2.17E-03 | 1.00E-04 | 7.54E-03 | 1.01E-03 |

**Table S1B** Comparisons of FDRs (BH) and P-values (P) for the independent datasets (BOMA, UTR, GAIN, and MSG) for top 27 SCZ associated pathways.

| **Description** | **BOMA-UTR** | | **BOMA** | | **UTR** | | **GAIN-MGS** | | **GAIN** | | **MSG** | |
| --- | --- | --- | --- | --- | --- | --- | --- | --- | --- | --- | --- | --- |
|  | **BH** | **P** | **BH** | **P** | **BH** | **P** | **BH** | **P** | **BH** | **P** | **BH** | **P** |
| dbMIR:gagcctg,mir-484 | **1.60E-02** | **1.32E-04** | **8.25E-03** | **7.34E-03** | 2.06E-01 | **4.72E-02** | **1.01E-04** | **6.66E-06** | **1.68E-03** | **2.49E-04** | **6.49E-03** | **1.15E-03** |
| dbMIR:gcaagac,mir-431 | **1.60E-02** | **3.86E-04** | **1.34E-02** | **1.29E-02** | 2.06E-01 | 7.62E-02 | 1.94E-01 | 1.44E-01 | 7.08E-02 | **3.67E-02** | 2.56E-01 | 2.11E-01 |
| dbGO:0008270:zinc ion binding | **1.58E-02** | **8.13E-06** | **4.99E-13** | **1.85E-14** | 3.85E-01 | 2.85E-01 | **1.01E-04** | **7.46E-06** | **9.06E-07** | **3.36E-08** | **8.24E-03** | **2.14E-03** |
| dbGO:0010628:positive regulation of gene expression | **2.34E-02** | **3.21E-04** | **5.69E-06** | **1.26E-06** | 2.93E-01 | 1.63E-01 | **7.88E-04** | **1.75E-04** | **3.79E-02** | **1.12E-02** | **2.74E-03** | **1.02E-04** |
| dbGO:0016564:transcription repressor activity | **1.55E-02** | **2.46E-05** | **1.65E-05** | **4.27E-06** | 1.74E-01 | **1.82E-02** | **2.00E-02** | **8.16E-03** | 1.34E-01 | 8.45E-02 | 1.45E-01 | 1.07E-01 |
| dbGO:0030528:transcription regulator activity | **1.55E-02** | **2.94E-05** | **2.38E-06** | **2.65E-07** | 2.93E-01 | 1.57E-01 | **5.42E-03** | **1.41E-03** | 2.08E-01 | 1.54E-01 | **1.11E-02** | **4.11E-03** |
| dbGO:0046914:transition metal ion binding | **5.17E-04** | **8.20E-07** | **2.22E-10** | **1.65E-11** | 3.67E-01 | 2.37E-01 | **1.02E-04** | **1.14E-05** | **1.23E-06** | **9.11E-08** | **6.49E-03** | **1.20E-03** |
| dbGO:0050808:synapse organization | **3.21E-02** | **2.89E-05** | **5.73E-04** | **2.99E-04** | 2.55E-01 | 1.23E-01 | 3.28E-01 | 2.92E-01 | 5.10E-01 | 4.53E-01 | **3.99E-02** | **1.92E-02** |
| dbKEGG:03013:rna transport | **3.20E-02** | **3.26E-03** | **6.92E-03** | **5.89E-03** | 4.39E-01 | 3.57E-01 | **3.90E-04** | **5.78E-05** | **6.82E-03** | **1.26E-03** | **4.74E-03** | **4.41E-04** |
| dbKEGG:04210:apoptosis | **1.97E-02** | **6.42E-04** | **1.58E-03** | **1.15E-03** | 2.06E-01 | 7.24E-02 | **3.33E-02** | **1.48E-02** | 1.84E-01 | 1.30E-01 | **4.55E-02** | **2.36E-02** |
| dbKEGG:04310:wnt signaling pathway | **8.32E-03** | **1.55E-04** | **9.02E-03** | **8.36E-03** | 3.67E-01 | 2.45E-01 | 3.07E-01 | 2.62E-01 | 5.07E-01 | 4.32E-01 | 5.30E-02 | **3.14E-02** |
| dbKEGG:04370:vegf signaling pathway | **7.23E-03** | **4.36E-05** | **4.36E-03** | **3.55E-03** | 4.52E-01 | 3.95E-01 | 7.69E-01 | 7.69E-01 | 8.50E-01 | 8.50E-01 | 5.25E-01 | 5.06E-01 |
| dbKEGG:04514:cell adhesion molecules (cams) | **2.02E-02** | **8.45E-04** | **7.87E-04** | **4.66E-04** | 3.32E-01 | 1.97E-01 | **1.21E-02** | **4.02E-03** | **4.22E-02** | **1.56E-02** | **4.74E-03** | **5.27E-04** |
| dbKEGG:04610:complement and coagulation cascades | **2.16E-02** | **1.40E-03** | **2.38E-02** | **2.38E-02** | 2.55E-01 | 1.17E-01 | 7.69E-01 | 7.60E-01 | 7.35E-01 | 7.08E-01 | 6.30E-01 | 6.30E-01 |
| dbRC:983189:kinesins | **5.82E-02** | **1.06E-04** | **1.80E-04** | **8.67E-05** | 2.06E-01 | **4.66E-02** | 2.31E-01 | 1.79E-01 | 6.57E-01 | 6.08E-01 | 1.06E-01 | 7.46E-02 |
| dbCGP:Kyng dna damage by uv | **2.89E-02** | **9.89E-05** | **1.05E-03** | **7.03E-04** | **4.96E-02** | **1.84E-03** | 1.49E-01 | 9.96E-02 | **2.57E-02** | **6.65E-03** | 5.30E-02 | **3.07E-02** |
| dbCGP:Lu aging brain up | **3.17E-02** | **2.95E-04** | **1.58E-03** | **1.17E-03** | 4.52E-01 | 4.02E-01 | 5.73E-01 | 5.30E-01 | 2.95E-01 | 2.40E-01 | 4.48E-01 | 3.98E-01 |
| dbCGP:Odonnell targets of myc and tfrc up | **4.70E-02** | **7.38E-04** | **3.61E-03** | **2.81E-03** | 1.94E-01 | **2.88E-02** | 3.07E-01 | 2.52E-01 | 7.08E-02 | **3.65E-02** | 5.25E-01 | 5.00E-01 |
| dbTFT:v$cebpa 01 | **4.03E-04** | **3.54E-06** | **8.78E-05** | **3.90E-05** | 7.73E-01 | 7.73E-01 | 7.84E-02 | **4.07E-02** | **1.07E-02** | **2.37E-03** | 7.67E-02 | **4.83E-02** |
| dbTFT:v$chop 01 | **3.76E-05** | **1.65E-07** | **8.73E-05** | **3.56E-05** | 3.85E-01 | 2.74E-01 | **5.51E-03** | **1.63E-03** | **3.86E-02** | **1.29E-02** | **6.59E-03** | **1.46E-03** |
| dbTFT:v$ciz 01 | **3.76E-05** | **1.63E-07** | **5.31E-06** | **9.84E-07** | 5.56E-01 | 5.15E-01 | **1.59E-02** | **5.88E-03** | 6.35E-02 | **2.82E-02** | **2.06E-02** | **9.14E-03** |
| dbTFT::v$hnf4 q6 | **5.85E-04** | **6.42E-06** | **3.93E-05** | **1.31E-05** | 2.06E-01 | 6.79E-02 | **5.04E-04** | **9.33E-05** | **1.68E-03** | **1.96E-04** | **1.11E-02** | **3.76E-03** |
| dbTFT:v$hp1sitefactor q6 | **1.18E-03** | **3.63E-05** | **4.96E-06** | **7.36E-07** | 4.18E-01 | 3.25E-01 | 1.68E-01 | 1.18E-01 | 1.69E-01 | 1.13E-01 | 2.56E-01 | 2.18E-01 |
| dbTFT:v$irf1 01 | **4.03E-04** | **2.66E-06** | **8.71E-04** | **5.48E-04** | 1.74E-01 | **1.93E-02** | 7.89E-02 | **4.56E-02** | 1.24E-01 | 6.89E-02 | 8.06E-02 | 5.37E-02 |
| dbTFT:v$ptf1beta q6 | **1.02E-03** | **1.60E-05** | **1.71E-05** | **5.08E-06** | 2.06E-01 | 5.47E-02 | 1.07E-01 | 6.73E-02 | 2.20E-01 | 1.71E-01 | **1.34E-02** | **5.45E-03** |
| dbTFT:v$sox5 01 | **1.02E-03** | **2.01E-05** | **7.27E-05** | **2.69E-05** | 2.43E-01 | 9.91E-02 | 5.32E-02 | **2.56E-02** | 1.34E-01 | 8.08E-02 | **1.11E-02** | **3.63E-03** |
| dbTFT:v$yy1 01 | **1.25E-03** | **5.22E-05** | **5.73E-04** | **3.18E-04** | 5.65E-01 | 5.44E-01 | 7.89E-02 | **4.68E-02** | 5.91E-02 | **2.41E-02** | 2.22E-01 | 1.73E-01 |

Note: bold – significant (cutof vlaue of 0.05)

**Table S1C** Comparisons of FDRs (BH) and p-values (P) for BOMA-UTR dataset for top 14 replicated SCZ associated pathways identified by different analysis methods.

| **Description** | **Global Test** | | **ALIGATOR** | **GRASS** | **gseaSNP** |
| --- | --- | --- | --- | --- | --- |
|  | **BH** | **P** | **P** | **P** | **P** |
| dbCGP:Kyng dna damage by uv | 2.89E-02 | 9.89E-05 | 5.82E-02 | 6.09E-01 | 1.44E-01 |
| **dbGO:0008270:zinc ion binding** | **1.58E-02** | **8.13E-06** | **6.27E-01** | **1.90E-02** | **6.29E-01** |
| **dbGO:0010628:positive regulation of gene expression** | **2.34E-02** | **3.21E-04** | **5.17E-01** | **4.70E-02** | **8.14E-01** |
| **dbGO:0046914:transition metal ion binding** | **5.17E-04** | **8.20E-07** | **5.69E-01** | **2.20E-02** | **4.62E-01** |
| dbGO:0050808:synapse organization | 3.21E-02 | 2.89E-05 | 3.80E-01 | 2.68E-01 | 4.98E-01 |
| **dbKEGG:04210:apoptosis** | **1.97E-02** | **6.42E-04** | **4.04E-01** | **3.60E-02** | **6.70E-02** |
| dbKEGG:04514:cell adhesion molecules (cams) | 2.02E-02 | 8.45E-04 | 9.81E-01 | 4.99E-01 | 8.00E-01 |
| dbMIR:gagcctg,mir-484 | 1.60E-02 | 1.32E-04 | 1.87E-01 | 4.38E-01 | 5.67E-02 |
| **dbTFT::v$hnf4 q6** | **5.85E-04** | **6.42E-06** | **2.96E-01** | **1.70E-01** | **4.56E-02** |
| dbTFT:v$cebpa 01 | 4.03E-04 | 3.54E-06 | 7.79E-01 | 6.60E-02 | 3.05E-01 |
| dbTFT:v$chop 01 | 3.76E-05 | 1.65E-07 | 2.77E-01 | 2.06E-01 | 2.30E-01 |
| **dbTFT:v$ciz 01** | **3.76e-05** | **1.63E-07** | **3.50E-02** | **1.21E-01** | **1.63E-02** |
| dbTFT:v$ptf1beta q6 | 1.02E-03 | 1.60E-05 | 2.96E-01 | 2.70E-01 | 2.39E-01 |
| dbTFT:v$sox5 01 | 1.02E-03 | 2.01E-05 | 9.88E-01 | 8.03E-01 | 9.47E-01 |
