# Supplementary material for: Integrated Pathway-Based Approach Identifies Association between Genomic Regions at CTCF and CACNB2 and Schizophrenia
Source: PLoS Genet. 2014 Jun 5;10(6):e1004345. doi: 10.1371/journal.pgen.1004345 (PMC4046913; doi:10.1371/journal.pgen.1004345)
Supplement: Table S2 — Comparison of redundancies in the subsets of the 6 pathway databases/gene-set collections. (DOC) [file pgen.1004345.s005.doc]

**Table S2** Comparison of redundancies in the subsets of the 6 pathway databases/gene-set collection.

|  | MIR | GO | KEGG | Reactome | CGP | TFT |
| --- | --- | --- | --- | --- | --- | --- |
| minimum | 0.00 | 0.00 | 0.00 | 0.00 | 0.00 | 0.00 |
| median | 4.49 | 5.29 | 2.86 | 0.00 | 8.01 | 7.00 |
| maximum | 30.00 | 87.54 | 33.33 | 0.00 | 31.25 | 22.69 |

The gene overlap among the 27 associated pathways is calculated. A self-overlap (of 100%) was excluded from calculating the mean and the maximum overlap.

Pathway databases and gene set collections: MIR – microRNA targets, GO – Gene Ontology, KEGG - Kyoto Encyclopedia of Genes and Genomes, CGP – Chemical and Genomic Perturbations, TFT – Transcription Factor Targets.
