# Supplementary material for: Integrated Pathway-Based Approach Identifies Association between Genomic Regions at CTCF and CACNB2 and Schizophrenia
Source: PLoS Genet. 2014 Jun 5;10(6):e1004345. doi: 10.1371/journal.pgen.1004345 (PMC4046913; doi:10.1371/journal.pgen.1004345)
Supplement: Table S3 — (A) Genes overlapping between the 14 replicated pathways in the BOMA-UTR dataset and (B) the GAIN-MGS dataset. (C) Single nucleotide polymorphisms overlapping between the 14 replicated pathways in the BOMA-UTR dataset and (D) the GAIN-MGS dataset. (DOC) [file pgen.1004345.s006.doc]

**Table S3 Table S3A** Genes overlapping between 14 replicated pathways in the BOMA-UTR dataset.

|  | GAGCCTG,MIR-484 | GO:0008270 | GO:0046914 | V$HNF4_Q6 | GO:0010628 | V$CHOP_01 | hsa04514 | V$CIZ_01 | hsa04210 | V$SOX5_01 | V$CEBPA_01 | V$PTF1BETA_Q6 | KYNG_DNA_DAMAGE_BY_UV | GO:0050808 |
| --- | --- | --- | --- | --- | --- | --- | --- | --- | --- | --- | --- | --- | --- | --- |
| GAGCCTG,MIR-484 | 60 | 18 | 18 | 1 | 7 | 5 | 2 | 3 | 0 | 4 | 0 | 1 | 0 | 0 |
| GO:0008270 | 18 | 1041 | 1041 | 16 | 121 | 18 | 0 | 21 | 2 | 24 | 19 | 18 | 2 | 5 |
| GO:0046914 | 18 | 1041 | 1195 | 16 | 124 | 19 | 0 | 21 | 4 | 25 | 20 | 18 | 2 | 5 |
| V$HNF4_Q6 | 1 | 16 | 16 | 105 | 12 | 10 | 0 | 8 | 2 | 6 | 13 | 5 | 0 | 1 |
| GO:0010628 | 7 | 121 | 124 | 12 | 541 | 16 | 4 | 21 | 6 | 25 | 25 | 18 | 4 | 9 |
| V$CHOP_01 | 5 | 18 | 19 | 10 | 16 | 121 | 0 | 13 | 2 | 14 | 13 | 18 | 1 | 1 |
| hsa04514 | 2 | 0 | 0 | 0 | 4 | 0 | 83 | 1 | 0 | 2 | 2 | 1 | 1 | 7 |
| V$CIZ_01 | 3 | 21 | 21 | 8 | 21 | 13 | 1 | 113 | 0 | 11 | 8 | 10 | 0 | 2 |
| hsa04210 | 0 | 2 | 4 | 2 | 6 | 2 | 0 | 0 | 52 | 0 | 1 | 2 | 2 | 0 |
| V$SOX5_01 | 4 | 24 | 25 | 6 | 25 | 14 | 2 | 11 | 0 | 133 | 13 | 10 | 1 | 3 |
| V$CEBPA_01 | 0 | 19 | 20 | 13 | 25 | 13 | 2 | 8 | 1 | 13 | 126 | 8 | 0 | 2 |
| V$PTF1BETA_Q6 | 1 | 18 | 18 | 5 | 18 | 18 | 1 | 10 | 2 | 10 | 8 | 101 | 0 | 0 |
| KYNG_DNA_DAMAGE_BY_UV | 0 | 2 | 2 | 0 | 4 | 1 | 1 | 0 | 2 | 1 | 0 | 0 | 16 | 1 |
| GO:0050808 | 0 | 5 | 5 | 1 | 9 | 1 | 7 | 2 | 0 | 3 | 2 | 0 | 1 | 47 |

**Table S3 Table S3B** Genes overlapping between 14 replicated pathways in the GAIN-MGS dataset.

|  | GAGCCTG,MIR-484 | GO:0008270 | GO:0046914 | V$HNF4_Q6 | GO:0010628 | V$CHOP_01 | hsa04514 | V$CIZ_01 | hsa04210 | V$SOX5_01 | V$CEBPA_01 | V$PTF1BETA_Q6 | KYNG_DNA_DAMAGE_BY_UV | GO:0050808 |
| --- | --- | --- | --- | --- | --- | --- | --- | --- | --- | --- | --- | --- | --- | --- |
| GAGCCTG,MIR-484 | 86 | 20 | 20 | 3 | 8 | 5 | 2 | 5 | 0 | 4 | 0 | 2 | 1 | 0 |
| GO:0008270 | 20 | 1650 | 1650 | 20 | 160 | 22 | 1 | 26 | 4 | 32 | 22 | 24 | 2 | 6 |
| GO:0046914 | 20 | 1650 | 1885 | 20 | 163 | 24 | 1 | 26 | 6 | 34 | 23 | 24 | 2 | 6 |
| V$HNF4_Q6 | 3 | 20 | 20 | 166 | 17 | 13 | 1 | 9 | 2 | 7 | 13 | 8 | 0 | 1 |
| GO:0010628 | 8 | 160 | 163 | 17 | 773 | 21 | 4 | 26 | 13 | 33 | 27 | 23 | 4 | 11 |
| V$CHOP_01 | 5 | 22 | 24 | 13 | 21 | 158 | 0 | 14 | 3 | 17 | 15 | 18 | 1 | 1 |
| hsa04514 | 2 | 1 | 1 | 1 | 4 | 0 | 117 | 1 | 0 | 2 | 3 | 1 | 1 | 8 |
| V$CIZ_01 | 5 | 26 | 26 | 9 | 26 | 14 | 1 | 148 | 0 | 14 | 11 | 14 | 1 | 3 |
| hsa04210 | 0 | 4 | 6 | 2 | 13 | 3 | 0 | 0 | 78 | 2 | 1 | 4 | 2 | 0 |
| V$SOX5_01 | 4 | 32 | 34 | 7 | 33 | 17 | 2 | 14 | 2 | 179 | 17 | 13 | 1 | 3 |
| V$CEBPA_01 | 0 | 22 | 23 | 13 | 27 | 15 | 3 | 11 | 1 | 17 | 160 | 9 | 0 | 2 |
| V$PTF1BETA_Q6 | 2 | 24 | 24 | 8 | 23 | 18 | 1 | 14 | 4 | 13 | 9 | 143 | 0 | 0 |
| KYNG_DNA_DAMAGE_BY_UV | 1 | 2 | 2 | 0 | 4 | 1 | 1 | 1 | 2 | 1 | 0 | 0 | 26 | 1 |
| GO:0050808 | 0 | 6 | 6 | 1 | 11 | 1 | 8 | 3 | 0 | 3 | 2 | 0 | 1 | 58 |

**Table S3 Table S3C SNPs overlapping between 14 replicated pathways in the BOMA-UTR dataset.**

|  | GAGCCTG,MIR-484 | GO:0008270 | GO:0046914 | V$HNF4_Q6 | GO:0010628 | V$CHOP_01 | hsa04514 | V$CIZ_01 | hsa04210 | V$SOX5_01 | V$CEBPA_01 | V$PTF1BETA_Q6 | KYNG_DNA_DAMAGE_BY_UV | GO:0050808 |
| --- | --- | --- | --- | --- | --- | --- | --- | --- | --- | --- | --- | --- | --- | --- |
| GAGCCTG,MIR-484 | 1658 | 518 | 519 | 50 | 292 | 303 | 42 | 213 | 4 | 181 | 6 | 69 | 20 | 0 |
| GO:0008270 | 518 | 25455 | 25453 | 603 | 4056 | 747 | 82 | 1245 | 46 | 1002 | 530 | 973 | 252 | 250 |
| GO:0046914 | 519 | 25453 | 29356 | 606 | 4101 | 773 | 109 | 1248 | 66 | 1022 | 572 | 991 | 257 | 250 |
| hsa03013 | 15 | 105 | 111 | 16 | 69 | 21 | 6 | 3 | 1 | 2 | 14 | 19 | 12 | 18 |
| V$HNF4_Q6 | 50 | 603 | 606 | 3450 | 404 | 326 | 14 | 302 | 30 | 245 | 530 | 170 | 0 | 152 |
| GO:0010628 | 292 | 4056 | 4101 | 404 | 13608 | 559 | 138 | 970 | 190 | 1430 | 673 | 1294 | 154 | 216 |
| GO:0030528 | 109 | 4107 | 4140 | 487 | 7525 | 746 | 105 | 844 | 117 | 1004 | 691 | 686 | 54 | 67 |
| V$CHOP_01 | 303 | 747 | 773 | 326 | 559 | 4365 | 15 | 647 | 34 | 446 | 381 | 850 | 3 | 176 |
| hsa04514 | 42 | 82 | 109 | 14 | 138 | 15 | 3562 | 79 | 0 | 99 | 269 | 37 | 86 | 726 |
| V$CIZ_01 | 213 | 1245 | 1248 | 302 | 970 | 647 | 79 | 4443 | 0 | 663 | 654 | 669 | 2 | 63 |
| GO:0016564 | 16 | 1779 | 1796 | 105 | 2749 | 105 | 20 | 275 | 26 | 410 | 217 | 175 | 0 | 20 |
| hsa04210 | 4 | 46 | 66 | 30 | 190 | 34 | 0 | 0 | 985 | 23 | 18 | 102 | 37 | 0 |
| V$SOX5_01 | 181 | 1002 | 1022 | 245 | 1430 | 446 | 99 | 663 | 23 | 5641 | 419 | 449 | 23 | 294 |
| V$CEBPA_01 | 6 | 530 | 572 | 530 | 673 | 381 | 269 | 654 | 18 | 419 | 4113 | 447 | 7 | 167 |
| V$PTF1BETA_Q6 | 69 | 973 | 991 | 170 | 1294 | 850 | 37 | 669 | 102 | 449 | 447 | 4864 | 6 | 8 |
| KYNG_DNA_DAMAGE_BY_UV | 20 | 252 | 257 | 0 | 154 | 3 | 86 | 2 | 37 | 23 | 7 | 6 | 577 | 86 |
| GO:0050808 | 0 | 250 | 250 | 152 | 216 | 176 | 726 | 63 | 0 | 294 | 167 | 8 | 86 | 3204 |

**Table S3 Table S3D** SNPs overlapping between 14 replicated pathways in the GAIN-MGS dataset.

|  | GAGCCTG,MIR-484 | GO:0008270 | GO:0046914 | V$HNF4_Q6 | GO:0010628 | V$CHOP_01 | hsa04514 | V$CIZ_01 | hsa04210 | V$SOX5_01 | V$CEBPA_01 | V$PTF1BETA_Q6 | KYNG_DNA_DAMAGE_BY_UV | GO:0050808 |
| --- | --- | --- | --- | --- | --- | --- | --- | --- | --- | --- | --- | --- | --- | --- |
| GAGCCTG,MIR-484 | 2332 | 712 | 712 | 88 | 488 | 409 | 64 | 291 | 8 | 296 | 7 | 84 | 11 | 0 |
| GO:0008270 | 712 | 34704 | 34704 | 764 | 5488 | 938 | 72 | 1538 | 48 | 1305 | 688 | 568 | 340 | 367 |
| GO:0046914 | 712 | 34704 | 40248 | 767 | 5580 | 975 | 113 | 1542 | 78 | 1337 | 732 | 585 | 343 | 367 |
| V$HNF4_Q6 | 88 | 764 | 767 | 4375 | 443 | 391 | 13 | 320 | 45 | 347 | 669 | 178 | 0 | 172 |
| GO:0010628 | 488 | 5488 | 5580 | 443 | 18006 | 640 | 165 | 1101 | 237 | 1624 | 801 | 1477 | 208 | 287 |
| GO:0030528 | 236 | 5169 | 5238 | 481 | 9967 | 840 | 116 | 893 | 138 | 1351 | 769 | 701 | 88 | 82 |
| V$CHOP_01 | 409 | 938 | 975 | 391 | 640 | 5436 | 6 | 781 | 45 | 551 | 396 | 952 | 7 | 177 |
| hsa04514 | 64 | 72 | 113 | 13 | 165 | 6 | 4846 | 117 | 0 | 138 | 414 | 44 | 109 | 861 |
| V$CIZ_01 | 291 | 1538 | 1542 | 320 | 1101 | 781 | 117 | 5987 | 0 | 806 | 737 | 749 | 5 | 60 |
| hsa04210 | 8 | 48 | 78 | 45 | 237 | 45 | 0 | 0 | 1304 | 31 | 17 | 105 | 34 | 0 |
| V$SOX5_01 | 296 | 1305 | 1337 | 347 | 1624 | 551 | 138 | 806 | 31 | 7679 | 616 | 564 | 23 | 328 |
| V$CEBPA_01 | 7 | 688 | 732 | 669 | 801 | 396 | 414 | 737 | 17 | 616 | 5133 | 348 | 9 | 188 |
| V$PTF1BETA_Q6 | 84 | 568 | 585 | 178 | 1477 | 952 | 44 | 749 | 105 | 564 | 348 | 5273 | 10 | 10 |
| KYNG_DNA_DAMAGE_BY_UV | 11 | 340 | 343 | 0 | 208 | 7 | 109 | 5 | 34 | 23 | 9 | 10 | 732 | 109 |
| GO:0050808 | 0 | 367 | 367 | 172 | 287 | 177 | 861 | 60 | 0 | 328 | 188 | 10 | 109 | 3862 |
