# Supplementary material for: Integrated Pathway-Based Approach Identifies Association between Genomic Regions at CTCF and CACNB2 and Schizophrenia
Source: PLoS Genet. 2014 Jun 5;10(6):e1004345. doi: 10.1371/journal.pgen.1004345 (PMC4046913; doi:10.1371/journal.pgen.1004345)
Supplement: Table S4 — List of schizophrenia (SCZ) associated genes, their p-values (FORGE analysis), and membership in the SCZ associated pathways discovered and replicated in the present study. Pathways in bold also showed an overall association using one of the other three methods (ALIGATOR, GRASS, gseaSNP) applied in the present study. (DOC) [file pgen.1004345.s007.doc]

|  | **HUGO** | **SIM_Z_FIX** | **pathways** |
| --- | --- | --- | --- |
| 1 | AKT3 | 6.52E-04 | **dbKEGG:04210:apoptosis** |
| 2 | CACNB2 | 8.57E-04 | dbGO:0050808:synapse organization,  dbTFT:v$chop_01,  dbTFT:v$cebpa_01,  **dbTFT:v$hnf4_q6** |
| 3 | MMP16 | 1.59E-03 | **dbGO:0046914:transition metal ion binding**,  **dbGO:0008270:zinc ion binding**,  **dbTFT:v$hnf4_q6** |
| 4 | WNT3 | 2.00E-03 | **dbGO:0010628:positive regulation of gene expression**,  **dbTFT:v$hnf4_q6** |
| 5 | FHIT | 2.20E-03 | **dbGO:0046914:transition metal ion binding** |
| 6 | GPC6 | 3.67E-03 | dbTFT:v$ptf1beta_q6 |
| 7 | TRIM8 | 4.32E-03 | dbTFT:v$cebpa_01 |
| 8 | NOS2 | 6.00E-03 | **dbGO:0046914:transition metal ion binding** |
| 9 | PELI2 | 8.50E-03 | dbTFT:v$sox5_01 |
| 10 | GRM3 | 9.20E-03 | **dbTFT:v$ciz_01** |
| 11 | CREB5 | 1.40E-02 | **dbGO:0010628:positive regulation of gene expression**,  **dbGO:0046914:transition metal ion binding**,  **dbGO:0008270:zinc ion binding** |
| 12 | ELAVL4 | 1.40E-02 | dbTFT:v$sox5_01 |
| 13 | CTCF | 1.50E-02 | **dbGO:0010628:positive regulation of gene expression**,  **dbGO:0046914:transition metal ion binding**,  **dbGO:0008270:zinc ion binding**,  dbTFT:v$ptf1beta_q6 |
| 14 | ZFYVE28 | 1.50E-02 | **dbGO:0046914:transition metal ion binding**,  **dbGO:0008270:zinc ion binding** |
| 15 | GTF3A | 2.55E-02 | **dbGO:0046914:transition metal ion binding**,  **dbGO:0008270:zinc ion binding** |
| 16 | PRDM10 | 2.90E-02 | **dbGO:0046914:transition metal ion binding**,  **dbGO:0008270:zinc ion binding** |
| 17 | NRXN1 | 3.45E-02 | dbGO:0050808:synapse organization,  dbKEGG:04514:cell adhesion molecules (cams) |
| 18 | LNX2 | 4.70E-02 | **dbGO:0046914:transition metal ion binding**,  **dbGO:0008270:zinc ion binding** |

**Supplementary Table S4** List of SCZ associated genes, their p-values (FORGE analysis) and membership in the SCZ associated pathways discovered and replicated in this study. Pathways are bold in case they show an overall association using also one of the other three methods (ALIGATOR, GRASS, gseaSNP) used in this study.
