# Supplementary material for: Integrated Pathway-Based Approach Identifies Association between Genomic Regions at CTCF and CACNB2 and Schizophrenia
Source: PLoS Genet. 2014 Jun 5;10(6):e1004345. doi: 10.1371/journal.pgen.1004345 (PMC4046913; doi:10.1371/journal.pgen.1004345)
Supplement: Table S7 — The Global Test results for the discovered gene-sets remained significant when the test was repeated with varying degrees of multicollinearity in the data. (DOC) [file pgen.1004345.s010.doc]

**Table S7** The Global Test results for the discovered gene-sets remained significant when the test was repeated with the varying degrees of collinearity in the data.

|  | pathway_IDs | **unpruned** | | **VIF100** | | **VIF10** | | **VIF5** | | **VIF2** | |
| --- | --- | --- | --- | --- | --- | --- | --- | --- | --- | --- | --- |
|  |  | **BH** | **P** | **BH** | **P** | **BH** | **P** | **BH** | **P** | **BH** | **P** |
| 1 | GAGCCTG,MIR-484 | 2.72E-04 | 2.12E-04 | 1.88E-04 | 1.32E-04 | 8.97E-05 | 4.98E-05 | 1.79E-04 | 6.26E-05 | 3.72E-04 | 8.28E-05 |
| 2 | GCAAGAC,MIR-431 | 2.65E-04 | 1.96E-04 | 4.74E-04 | 3.86E-04 | 4.82E-03 | 4.46E-03 | 2.99E-03 | 2.44E-03 | 1.33E-01 | **1.28E-01** |
| 3 | GO:0008270 | 6.30E-10 | 2.33E-11 | 3.26E-08 | 2.41E-09 | 4.25E-07 | 4.13E-08 | 9.11E-05 | 2.03E-05 | 1.43E-05 | 1.41E-06 |
| 4 | GO:0010628 | 9.39E-06 | 2.88E-06 | 1.26E-05 | 4.21E-06 | 2.92E-05 | 1.19E-05 | 2.73E-04 | 1.67E-04 | 3.86E-04 | 1.00E-04 |
| 5 | GO:0016564 | 1.75E-06 | 3.25E-07 | 3.02E-06 | 6.72E-07 | 1.44E-05 | 3.73E-06 | 2.73E-04 | 1.70E-04 | 3.81E-03 | 1.84E-03 |
| 6 | GO:0030528 | 8.19E-08 | 9.10E-09 | 1.05E-07 | 1.17E-08 | 9.74E-07 | 1.44E-07 | 1.81E-04 | 8.05E-05 | 4.83E-04 | 1.61E-04 |
| 7 | GO:0046914 | 9.76E-10 | 7.23E-11 | 1.87E-09 | 6.93E-11 | 5.66E-08 | 2.10E-09 | 4.91E-06 | 3.64E-07 | 1.26E-05 | 4.65E-07 |
| 8 | GO:0050808 | 9.39E-06 | 3.13E-06 | 3.59E-05 | 1.55E-05 | 2.22E-04 | 1.48E-04 | 2.73E-04 | 1.72E-04 | 4.56E-02 | 3.89E-02 |
| 9 | hsa03013 | 4.38E-03 | 4.21E-03 | 3.26E-03 | 3.26E-03 | 1.56E-02 | 1.56E-02 | 1.66E-01 | 1.66E-01 | 2.42E-01 | **2.42E-01** |
| 10 | hsa04210 | 2.61E-04 | 1.83E-04 | 7.54E-04 | 6.42E-04 | 1.96E-04 | 1.24E-04 | 8.48E-04 | 6.29E-04 | 2.52E-02 | 1.87E-02 |
| 11 | hsa04310 | 1.40E-04 | 8.26E-05 | 2.09E-04 | 1.55E-04 | 1.15E-03 | 9.35E-04 | 4.17E-03 | 3.55E-03 | 9.50E-04 | 3.52E-04 |
| 12 | hsa04370 | 2.70E-05 | 1.40E-05 | 7.85E-05 | 4.36E-05 | 3.14E-04 | 2.21E-04 | 2.46E-03 | 1.91E-03 | 1.24E-02 | 7.82E-03 |
| 13 | hsa04514 | 1.14E-03 | 1.06E-03 | 9.13E-04 | 8.45E-04 | 3.22E-03 | 2.86E-03 | 5.20E-03 | 4.81E-03 | 4.56E-02 | 3.78E-02 |
| 14 | hsa04610 | 7.73E-03 | 7.73E-03 | 1.46E-03 | 1.40E-03 | 2.05E-03 | 1.75E-03 | 4.90E-03 | 4.35E-03 | 3.60E-02 | 2.80E-02 |
| 15 | hsa983189 | 3.26E-04 | 2.65E-04 | 1.58E-04 | 1.06E-04 | 4.93E-03 | 4.75E-03 | 8.52E-03 | 8.21E-03 | 1.07E-02 | 6.37E-03 |
| 16 | KYNG_DNA_DAMAGE_BY_UV | 1.40E-04 | 8.82E-05 | 1.57E-04 | 9.89E-05 | 3.62E-04 | 2.68E-04 | 2.98E-04 | 2.10E-04 | 1.29E-01 | **1.19E-01** |
| 17 | LU_AGING_BRAIN_UP | 6.52E-04 | 5.55E-04 | 3.79E-04 | 2.95E-04 | 1.82E-04 | 1.08E-04 | 1.79E-04 | 4.70E-05 | 1.04E-03 | 4.24E-04 |
| 18 | ODONNELL_TARGETS_OF_MYC_AND_TFRC_UP | 7.92E-04 | 7.04E-04 | 8.31E-04 | 7.38E-04 | 7.31E-04 | 5.68E-04 | 2.98E-04 | 2.06E-04 | 9.31E-03 | 5.17E-03 |
| 19 | V$CEBPA_01 | 2.24E-05 | 9.97E-06 | 1.19E-05 | 3.53E-06 | 5.02E-06 | 1.11E-06 | 9.11E-05 | 1.92E-05 | 2.13E-02 | 1.50E-02 |
| 20 | V$CHOP_01 | 1.86E-06 | 4.13E-07 | 8.96E-07 | 1.66E-07 | 3.17E-06 | 5.86E-07 | 3.96E-05 | 4.40E-06 | 4.46E-04 | 1.32E-04 |
| 21 | V$CIZ_01 | 1.61E-06 | 2.38E-07 | 8.96E-07 | 1.63E-07 | 7.10E-05 | 3.42E-05 | 2.07E-04 | 9.95E-05 | 1.82E-03 | 8.08E-04 |
| 22 | V$HNF4_Q6 | 9.39E-06 | 2.97E-06 | 1.73E-05 | 6.42E-06 | 1.58E-05 | 4.68E-06 | 2.67E-04 | 1.38E-04 | 5.57E-02 | 4.95E-02 |
| 23 | V$HP1SITEFACTOR_Q6 | 1.40E-04 | 8.80E-05 | 7.01E-05 | 3.63E-05 | 2.11E-05 | 7.81E-06 | 1.79E-04 | 6.64E-05 | 3.51E-04 | 6.50E-05 |
| 24 | V$IRF1_01 | 2.07E-05 | 8.41E-06 | 1.03E-05 | 2.66E-06 | 4.25E-07 | 4.72E-08 | 1.86E-06 | 6.89E-08 | 1.43E-05 | 1.59E-06 |
| 25 | V$PTF1BETA_Q6 | 1.33E-05 | 4.92E-06 | 3.59E-05 | 1.60E-05 | 5.80E-05 | 2.58E-05 | 8.31E-05 | 1.23E-05 | 2.41E-04 | 3.57E-05 |
| 26 | V$SOX5_01 | 2.55E-05 | 1.23E-05 | 4.18E-05 | 2.01E-05 | 8.63E-05 | 4.47E-05 | 1.79E-04 | 5.39E-05 | 8.32E-03 | 4.32E-03 |
| 27 | V$YY1_01 | 2.61E-04 | 1.81E-04 | 8.81E-05 | 5.22E-05 | 1.86E-05 | 6.19E-06 | 1.81E-04 | 7.91E-05 | 1.87E-02 | 1.25E-02 |
