# Supplementary material for: Integrated Pathway-Based Approach Identifies Association between Genomic Regions at CTCF and CACNB2 and Schizophrenia
Source: PLoS Genet. 2014 Jun 5;10(6):e1004345. doi: 10.1371/journal.pgen.1004345 (PMC4046913; doi:10.1371/journal.pgen.1004345)
Supplement: Text S1 — Description of supplementary results and methods. (DOC) [file pgen.1004345.s011.doc]

**Supplementary Results and Methods**

**SUPPLEMENTARY RESULTS**

**Application of alternative pathway analysis approaches**. As an alternative to the Global Test, three other algorithms described in the literature were applied to the present data: (i) a regularized approach, GRASS [1]; (ii) gseaSNP [2]; and (iii) ALIGATOR [3] as implemented in the R package “SNPath” [1]. In each case, one thousand permutations were performed in order to calculate the empirical null distribution of the test statistic. Logistic regression was used to calculate the individual single nucleotide polymorphism (SNP) association statistics. For the performance of ALIGATOR, SNPs with p-values of < 0.05 were selected.

Of the 14 replicated pathways, six were significantly associated with schizophrenia in the discovery sample (BOMA-UTR) in at least one of GRASS [1], ALIGATOR [3], and gseaSNP [2] (Table ***S1C***). These six pathways comprise: (i) the three Gene Ontology (GO) terms: zinc ion binding, transition metal ion binding, and positive regulation of gene expression; (ii) two transcription factor targets gene-sets; and (iii) the apoptosis pathways described in the Kyoto Encyclopedia of Genes and Genomes (KEGG) database.

**SUPPLEMENTARY METHODS**

**Mapping SNPs to genes**. SNPs were annotated using information from dbSNP Build 127. The “seq-gene” file was downloaded from the NCBI ftp website (BUILD.36.3). The following six columns were extracted for the “feature_type” of “GENE”: feature_id; feature_type; chromosome; chr_start; chr_stop; and chr_orient. The SNP rs number and its chromosomal location from the dbSNP chromosome report file were compared to the extracted „seq-gene“ file according to first chromosome and then to position. SNP rs numbers, chromosome number, chromosome position, contig accession number, and ENTREZ gene IDs in the regions which these SNPs lie were obtained.

**SNP label permutation test**. A permuted dataset was generated by shuffling SNP labels in the entire set of SNPs. The association statistics for each pathway were re-calculated in each permutation using the Global Test. To obtain the null distribution of test statistics for each pathway, one thousand permutations were drawn (each without replacement). The empirical p-value was calculated as the ratio of the number of permuted test statistics greater or equal to the unpermuted test statistic (observed test statistic in the original data) to the overall number of permutations. To remain conservative, one was added to the total number of permutations in order to avoid “p-values of zero, which occur when the test statistic is never surpassed by the permutation values” [4].

**Pathway overlap**. To visualize the relationships between the pathways of interest, a hierarchical clustering algorithm (using average linkage method with the Jaccard similarity coefficient as the distance measure) was used to assemble pathways with similar implicated SNP content in the BOMA-UTR dataset (***Figure S2***). An implicated SNP was defined as a SNP with an individual SNP p-value of ≤ 0.05. The overall gene and SNP overlap between all pairs of the replicated pathways are shown in Table S3***A*** and ***Table S3C*** respectively. For the GAIN-MGS dataset, information concerning gene and SNP overlap is provided in Table S3***B*** and ***Table S3D****,* respectively.

**Selection of pathways and gene-sets.** A usual prerequisite of pathway association analysis is the selection of gene-set collections that reflect the nature of complex disorders, i.e. the potential involvement of a diverse set of biological functions. The gene-set collections tested in the present study were therefore organized around heterogeneous data models which aim to fulfil this criterion. The following collections were assessed: four collections of canonical pathways, i.e. BioCarta, Kyoto Encyclopedia of Genes and Genomes (KEGG), Reactome, and Chemical and Genetic Perturbations (CGP); two motif gene-set collections i.e. microRNA Targets (MIR) and Transcription Factor Targets (TFT); and Gene Ontology terms (GO). KEGG is one of the most widely used databases of this nature, and includes pathways involved in cellular processes, drug response, environmental-information-processing, genetic-information-processing, human diseases, and metabolism. Reactome includes peer-reviewed metabolic and signalling pathways, which are presented in an experimentally confirmed or inferred reaction-centred form. The CGP gene-sets were used to test the hypothesis that individuals with schizophrenia have different SNP profiles in sets of genes that represent expression signatures of chemical and genetic perturbations (such as deletions, insertions, or frame shift mutations), compared to controls. In the case of the motif gene-sets, we focused on SNPs that overlap (according to experimentally detected or computationally predicted data) with transcription factor binding sites or micro-RNA target sites. The GO gene-set collections facilitate the development of new hypotheses concerning the biological processes, cellular components, and molecular functions that underlie complex diseases*.*

Pathway information is, at best, incomplete, and is expected to alter / expand as new knowledge concerning certain biological processes is gained. Moreover, recent studies have suggested that certain information in the most widely used pathway databases is out-dated [6], incomplete, or missing [5]. One way of overcoming the incompleteness of pathway information is to combine information concerning nominally identical pathways from different databases. However the resulting pathway information may be ambiguous, since closely related, or even nominally identical, pathways contain sets of genes with minimal gene overlap. This is due to the fact that each database is constructed and assembled according to different data models and conventions. These inconsistencies in pathway composition suggest that the most comprehensive results will be obtained by performing separate analyses for each individual database.

Based on the report by Vivar et al. [7], we expected an average redundancy for the information in pathways described in KEGG, BioCarta, CGP, and Reactome of between 3.86 - 21.24%. For the databases listed above, the percentage of pathways with complete overlap (100%) was expected to be between 0.01-0.6%. The level of redundancy between each pair of pathways included in the present 27 pathways/gene-sets was calculated, and this information is provided in Figure S1 and Table S3.

Pathways were not excluded on the basis of overlapping gene content for two reasons. Firstly, as mentioned above, we observed that a number of genes in nominally identical pathways differed between different sources. For instance, the MAPK signaling pathway description in BioCarta includes 87 genes, whereas KEGG assigns 267 genes to this pathway. Moreover, only a 25% overlap between these two gene-sets is apparent. Secondly, for pathways with high gene overlap, several approaches have been proposed for reducing multiplicity through collapsing sets of highly redundant pathways into embedded pathways [7]. However, selection of a threshold for collapsing pathways into a one super-pathway on the basis of a reasonable level of overlap is arbitrary, and has unforeseeable consequences in terms of the tested hypothesis. For example, although combining pathways with at least 75% overlap might produce a moderate reduction in multiplicity [7], this approach results (at least partly) in super-pathways that reflect related, but truly diverse, biological mechanisms.

The multiple testing burden was therefore reduced using a two step approach. Firstly, prior to pathway analysis, gene sets that were too small (less than 5 genes) or too large (more than 200 genes) were eliminated, a procedure that is widely accepted in the field. In the discovery stage of the pathway association analysis, separate correction for multiple testing was then applied to each set of pathways. Secondly, replication of our findings was attempted in independent samples, with correction for the number of tests performed. Employment of this two-step approach resulted in a correction that controlled for the number of tests performed, while reflecting the related nature of the gene-set collections that were accessed.

**SNP independence and Linkage disequlibrium-based SNP pruning.** One of the key assumptions of logistic regression is that there is little multicollinearity (as measured by the variation inflation factor, VIF) among variables. However, in practice, many SNPs are correlated with each other. The literature includes varying recommendations for the use of VIFs in order to ensure a sufficiently small level of multi-collinearity. For example, a VIF of 10 was suggested by Hair et al. [8], a VIF of 5 by Rogerson [9], and a VIF of 4 by Pan and Jackson [10]. Backhaus et al. [11] suggested that VIF of 5 and above might suggest multicollinearity and according to Fahrmeir et al. [12], a VIF of above 100 should be viewed as a degree of evidence for multicollinearity [13].

Since removing SNPs on the basis of a strict VIF threshold may remove biologically relevant information from the data [14], we controlled for collinearity at two points in the analyses. Firstly, prior to pathway analysis, the SNP set was pruned using a VIF threshold of 100. Secondly, after the exploratory stage of the pathway analysis and for all sets of SNPs representing significant pathways, an iterative pruning was applied, based on a VIF threshold of 100. This second pruning procedure involved calculating a matrix of VIFs for all SNPs annotated to these pathway, and then removing one SNP of the SNP pair with the highest VIF in this matrix at random. This procedure was repeated until all VIFs in the matrix were below 100. The resulting SNPs (with VIFs of below 100) were then used to recalculate the Global Test score using explanatory variables with less collinearity. We also compared results obtained with a VIF threshold of 100 with results obtained for VIF thresholds of 10, 5, and 2 (Table S7). It is of note that the vast majority of the 27 pathways remained significant after application of the strongest VIF threshold in our comparison (VIF = 2). Only three pathways were non-significant, and all three still showed a trend towards association, with no p-value of greater than 0.25 being observed.

**Subject vs SNP label permutations.** A factor which may introduce bias in pathway association analyses is pathway size, i.e. the number of SNPs that are mapped to the pathway. For small pathways, the results may be influenced by single-SNP effects, and for larger pathways, chance associations may be observed. To control this bias, we first constrained our analysis on pathway size at the gene-level, i.e. only gene-sets with at least 5 and at most 200 genes were included. This constraint was not applied to the GO terms due to their nested structure. Additional steps were undertaken to minimize the impact of other potential biases. The SNP-label permutation without replacement was performed to correct for bias due to different pathways and consisted of (i) different numbers of genes and (ii) different genes with different numbers of mapped SNPs. The upper bound p-values [4] were calculated with 1000 permutations [15]. The advantages of this method are two-fold: (i) as the number of permutations decreases, the test becomes more conservative, (ii) skewed test results from the multiple testing correction procedures are avoided, since no p-values of zero will be obtained [4]. A significant p-value from the SNP-label permutation test indicates a low probability of obtaining a test statistic value more extreme than that observed when the test is performed with the randomly selected SNPs of the same size. The SNP-label permutation test was coupled with the subject-sampling test, as recommended by Efron and Tibshirani [16]. The subject-sampling permutation was performed to test whether pathway analysis was biased due to a random variation at the individual level. The case-control status was randomized 10,000 times. The significant p-values from this test indicate that the same pathway associations will be found in an independent cohort.

The p-values obtained from these two tests were compared (Figure S3). For the 215 KEGG pathways, 30 were significant after correction of p-values for multiple testing using a subject-sampling method. Of these 30 pathways, one failed the SNP-label permutation test. (The significant disease pathways were cancer related and were removed from the final list of discovered pathways.) Figure S3 demonstrates that many of the TFT gene-sets with more than 3000 SNPs were non-significant after the SNP-label permutation test, even though the subject-sampling p-values were significant. This indicates the necessity of employing both sampling tests in order to decrease the potential for false positive findings. This comparison did not include the gene-sets from BioCarta and GO terms, since for the former, no significant pathway was selected in the final discovered pathways, and for the latter, the SNP-label permutation test was not performed due to the nested structure of GO terms.

References

1. Chen LS, Hutter CM, Potter JD, Liu Y, Prentice RL, et al. (2010) Insights into Colon Cancer Etiology via a Regularized Approach to Gene Set Analysis of GWAS Data. Am J Hum Genet, 86: 860-871.
2. Wang K, Li M, Bucan M (2007) Pathway-Based Approaches for Analysis of Genomewide Association Studies. Am J Hum Genet 81: 1278–1283.

# Holmans P, Green EK, Pahwa JS, Ferreira MA, Purcell SM, et al. (2009) Gene ontology analysis of GWA study datasets provides insights into the biology of bipolar disorder. Am J Hum Genet 85: 13-24.

1. Phipson B, Smyth G (2010) Permutation p-values should never be zero: Calculating exact p-values when permutations are randomly drawn. Stat Appl Genet Mol Biol 9: 39.
2. Ooi HS, Schneider G, Lim TT, Chan YL, Eisenhaber B, Eisenhaber F. (2010) Biomolecular pathway databases. Methods Mol Biol 609: 129-144.
3. Adriaens ME, Jaillard M, Waagmeester A, Coort SL, Pico AR, Evelo CT (2008) The public road to high-quality curated biological pathways. Drug Discov Today 13: 856–862.
4. Vivar JC, Pemu P, McPherson R, Ghosh S (2013) Redundancy Control in Pathway Databases (ReCiPa): An application for improving gene-set enrichment analysis in Omics studies and "Big Data" biology. OMICS A Journal of Integrative Biology 17: 414-422.
5. Hair JFJr, Anderson RE, Tatham RL, Black WC (1995) Multivariate Data Analysis (3rd ed). New York: Macmillan.
6. Rogerson PA (2001) Statistical Methods for Geography: A Student’s Guide. pp 337. London: Sage.
7. Pan, Y, Jackson, RT (2008) Ethnic difference in the relationship between acute inflammation and and serum ferritin in US adult males. Epidemiology and Infection, 136: 421-431.
8. Backhaus K, Erichson B, Plinke W, Weiber R (2003) Multivariate Analysemethoden – Eine anwendungsorientierte Einführung. 10th ed. Berlin: Springer.
9. Fahrmeir, L, Künstler, R, Pigeot, I, Tutz, G. (1999). Statistik. Der Weg zur Datenanalyse. 2nd ed. Berlin: Springer.
10. Hofmann JV (2009) Family Mindset as Predictor of Entrepreneurship in German Family Firms. PhD. Thesis, University of St. Galllen, Switzerland.
11. O'Brien RM (2007) A caution regarding rules of thumb for variance inflation factors. Quality & Quantity 41: 673-690.
12. Manly BFJ (1997) Randomization, Bootstrap and Monte Carlo Methods in Biology, 2nd edn. London: Chapman & Hall.
13. Efron B, Tibshirani R (2007) On testing the significance of sets of genes. Ann Appl Stat 1: 107-129.
